# Supplementary material for: Climate change impacts on the extreme power shortage events of wind-solar supply systems worldwide during 1980–2022
Source: Nat Commun. 2024 Jun 18;15:5225. doi: 10.1038/s41467-024-48966-y (PMC11189577; doi:10.1038/s41467-024-48966-y)
Supplement: Supplementary file 1 — Supplementary Information [file 41467_2024_48966_MOESM1_ESM.pdf]

Supplementary Information for

Climate change impacts on the power shortage events of wind-solar supply systems worldwide during 1980–2022

Dongsheng Zheng[1], Dan Tong[1*], Steven J. Davis[2,3], Yue Qin[4], Yang Liu[1], Ruochong Xu[1], Jin Yang[1], Xizhe Yan[1], Guannan Geng[5], Huizheng Che[6], Qiang Zhang[1]

[1]Ministry of Education Key Laboratory for Earth System Modeling, Department of Earth System Science, Tsinghua University, Beijing, 100084, China.

[2]Department of Earth System Science, University of California, Irvine, Irvine, CA 92697, USA.

[3]Department of Civil and Environmental Engineering, University of California, Irvine, Irvine, CA 92697, USA.

[4]College of Environmental Science and Engineering, Peking University, Beijing, 100871, China.

[5]State Key Joint Laboratory of Environment Simulation and Pollution Control, School of Environment, Tsinghua University, Beijing, People's Republic of China.

[6]State Key Laboratory of Severe Weather & Key Laboratory of Atmospheric Chemistry of CMA, Chinese Academy of Meteorological Sciences, Beijing, 100081, China.

*****Corresponding author: dantong@tsinghua.edu.cn

Supplementary Information:

Supplementary Notes 1–12
Supplementary Figures 1–51
Supplementary Tables 1–7

Supplementary Note 1. Sensitive tests of ERA5 reanalysis data on extreme power shortage events

Given the potential uncertainty of climatic variables from one-single weather data product (i.e., MERRA-2), it is necessary to verify our results using another independent reanalysis data. To address such a concern, we downloaded climatological data (including surface solar radiation downwards, top net solar radiation-clear sky, 100m u-component of wind speed, 100m v-component of wind speed, as well as 2m temperature) from ERA5 reanalysis product (https://cds.climate.copernicus.eu/cdsapp#!/home). We applied the same methods with MERRA-2 to calculate the hourly capacity factors of wind and solar power at a gridded scale, and then the gridded results were aggregated to estimate area-weighted average wind and solar capacity factors for individual countries. Afterwards, the resulting capacity factors were used to estimate system reliability and potential extreme power shortage events.

Supplementary Figure 2 shows the characteristics of defined extreme long-duration events for wind-solar supply systems during the period 1980–2022 using ERA5 reanalysis data products. Globally, wind-solar supply systems have experienced an increasing trend in extreme long-duration events, although there are repeated up and down undulations irrespective of their frequency, duration, and intensity. Long-duration events worldwide increased significantly at a rate of 0.031 per year in frequency (P<0.001; Supplementary Figure 3a), 0.322 hours per year in duration (Supplementary Figure 3b), and 0.120 per year in intensity (Supplementary Figure 3c). In particular, we find that extreme long-duration events over the last two decades considerably outnumbered those over the first two decades. For example, the annual average duration of extreme long-duration events rose evidently from 141.0 hours during 1980–2000 to 148.5 hours during 2001–2022 (one-way analysis of variance (ANOVA), P<0.001; Supplementary Figure 3b).

We further ranked the annual average frequency, duration, and intensity of extreme long-duration events over the past 43 years (Supplementary Figure 3d–f). Our results revealed that the greatest frequency, duration, and intensity of extreme long-duration events occurred intensively in the period between 2001 and 2022. For instance, the period from 2001 to 2022 accounted for 85% of the top 20 frequent years of extreme long-duration events (Supplementary Figure 3d). This case is even more apparent for the top 10 frequent years, 90% of which emerged over the period after 2000 (Supplementary Figure 3d). Moreover, the highest years for the three metrics are different (i.e., 2022 for frequency, 2013 for duration, and 2020 for intensity), which in turn suggests potential complexity in dispatching flexible electricity sources to defend against extreme power shortage events.

Spatial analyses further illustrated that the observed uptrend in extreme long-duration events is a global-scale phenomenon (Supplementary Figure 4). There are most countries with growing extreme long-duration events during the period of 1980–2000 and 2001–2022. In fact, there

over two-third of surveyed 178 countries with uptrends in frequency, duration, intensity of extreme long-duration events, respectively (Supplementary Figure 4). However, this ascending trend in extreme long-duration events at the global scale masks large spatial disparities across various countries. Indeed, the change in the frequency of extreme long-duration events peaked at +3.7 in Costa Rica, while bottoming at only –1.3 hours in Venezuela (Supplementary Figure 4h).

Supplementary Figure 5 shows the change in extreme low-reliability power events between 1980–2000 and 2001–2022 based upon ERA5 data. Unfortunately, extreme low-reliability events also followed a significant increasing trend over the past four decades. In the last two decades, extreme low-reliability events have increased by 1.0 (0.050 per year) in frequency (Supplementary Figure 5a), 6.7 hours (0.253 hours per year) in duration (Supplementary Figure 5b), and 2.3 (0.077 per year) in intensity (Supplementary Figure 5c) relative to the counterpart in the first two decades ($P<0.01$). Similar to extreme long-duration events, despite the global upwards trend in extreme low-reliability events, there are considerable discrepancies across different countries. For instance, the increase in the duration of extreme low-reliability events reached up to 124.9 hour in Indonesia, but only grew by 20.9 hours in Japan and even decreased by –3.4 hours in Tunisia (the second column in Supplementary Figure 5d).

We further explored the relationship of the two types of extreme power shortage events for 178 countries, most of which presented the same trends for the two types of extreme power shortage events (Supplementary Figure 6). Moreover, changes in extreme power shortage events are unevenly distributed but rather exhibit a latitudinal gradient and an evident disparity in countries with different economic development levels (Supplementary Figure 6). Specifically, developed countries in high latitudes generally show slight variability in extreme power shortage events, yet developing countries in low and middle latitudes tend to have drastic changes in extreme power shortage events. For example, the greatest changes in frequency of extreme power shortage events have emerged in developing countries in low- and middle-latitude countries (e.g., Nepal, Honduras and Costa Rica; blue bubbles at the edge of quadrants in Supplementary Figure 6a); however, high-latitude countries generally exhibit a small variability in frequency of extreme power shortage events.

In addition, we revealed climatological drivers underlying the detected growing extreme power shortage events by combining extremely low wind speed and solar radiation using ERA5 data (Supplementary Figure 7). We find that, the growth in extreme shortage events is largely attributable to prolonged extremely low wind speed (change in colors) and solar radiation (change in size) during the period between 1980–2000 and 2001–2022. In addition, there are large disproportionalities between changes in extreme power shortage events and variabilities in compound extremely low wind speed and solar radiation events. Small changes in annual average wind speed and solar radiation generally indicate considerable changes in annual hours

of compound extremely low wind speed and solar radiation events, even resulting in a larger variability in extreme power shortage events. In fact, even though the change in annual average wind speed and solar radiation across the 178 countries is as low as 1.62% (the pink circle in Supplementary Figure 7d), change in annual hours of compound extremely low wind speed and solar radiation events reach 9.55% (the blue circle in Supplementary Figure 7d–e). Moreover, such nearly 10% change in compound extremely low wind speed and solar radiation events results in up to average 21.37% change in in extreme long-duration and low-reliability power shortage events (the red circle in Supplementary Figure 7e).

Supplementary Note 2. Decadal analyses for extreme power shortage evets of wind-solar supply systems

The observed extreme long-duration events showed much more complicated decadal changes, with no significant disparities between 1980–1990 and 1991–2000 ($P<0.05$), but obvious uptrends regardless of its frequency, duration, and intensity between 1991–2000 and 2001–2010 (Supplementary Figures 8–10). We further ranked the annual average frequency, duration, and intensity of extreme long-duration events over the past 43 years (Supplementary Figure 8d–f). Our results revealed that the greatest frequency, duration, and intensity of extreme long-duration events occurred intensively in the period of 2001–2022 (the period of 2011–2022 in particular). For instance, the period from 2001 to 2022 accounted for 90% of the top 20 frequent years of extreme long-duration events, and the period of 2011 – 2022 occupied 60% (Supplementary Figure 8d). This case is even more apparent for the top 10 frequent years, all of which emerged over the period after 2000 and 70% of which occurred during the period after 2011 (Supplementary Figure 8d).

We further illustrated that the spatial pattern of the observed change in extreme long-duration events since 1980s (Supplementary Figure 9). The number of countries with increasing extreme long-duration events is usually higher than that of countries with decreasing extreme long-duration events. For example, there are 64% countries with uptrends in frequency of extreme long-duration events during the periods between 1990 and 2000. However, such upward trends in extreme long-duration events at the global scale mask large spatial disparities across various countries across the past four decades. For example, duration of extreme shortage events represented the highest increase by +106 hours in Chad between 2001–2010 and 2011–2022, but the largest decrease by in Seychelles between 1991–2000 and 2001–2010 (Supplementary Figure 9).

Apart from extreme long-duration events during, extreme low-reliability events are also severe challenges for maintaining wind-solar system stability. There are 168 out of 178 countries where extreme low-reliability events have emerged since 1980, except for countries with comparatively large land areas, such as Russia and China, as a result of their strong spatial aggregations and complementarities[1] (Supplementary Figures 14–15). Supplementary Figure

10 shows the change in extreme low-reliability power events during the past four decades. Unfortunately, extreme low-reliability events also followed a significant increasing trend over the past four decades. For example, duration of extreme low-reliability power shortage events over the periods between 1980–1990 and 1991–2000 have increased by 0.19.

Similar to extreme long-duration events, despite the global upwards trend in extreme low-reliability events, there are considerable discrepancies across different countries across the past four decades. For instance, the variability in frequency of extreme long-duration events ranged from nearly +13 in Benin between 1991–2000 and 2001–2010 to only around –12 in Suriname between 2001–2010 and 2011–2022 (Supplementary Figure 10). However, most countries experienced growth in either frequency, duration, and/or intensity of extreme low-reliability events on a decadal scale (Supplementary Figure 10). For example, there are 52% countries with growing intensity of low-reliability events during between 1980–1990 and 1991–2000. This case is even more evident between 2001–2010 and 2011–2022, for which 63% countries evinced uptrend in intensity of low-reliability events (Supplementary Figure 10i).

Supplementary Note 3. Potential drivers of changes in wind and solar output potentials

The observed growth in extreme shortage events results largely from prolonged extremely low wind speed and solar radiation. Globally, highly frequent extreme power shortage events in the recent period (e.g., 2010, 2022, and 2018) have resulted from comparatively long hours of extremely low wind speed or solar radiation, while a lower frequency of extreme power shortage events before 2000 (e.g., 1990 and 1994) is ascribed to shorter periods of extremely low wind speed or solar radiation (Supplementary Figure 23). The increases in extremely low wind speed and solar radiation are in accordance with significant declines in the annual mean wind speed and solar radiation since the 1980s ($P<0.01$; Supplementary Figure 19). These changes also conform with the increases in extremely low wind and solar capacity factors and the decreases in annual mean wind and solar capacity factors ($P<0.05$; Supplementary Figure 20). This relationship occurs mainly because wind speed and solar radiation predominate wind and solar capacity factors, respectively[2,3], although air density and ambient temperature still have weak interactive effects (Supplementary Figure 21).

Potential drivers behind the observed downtrend in wind power potential are highly complex and even remain contested. Some studies[4,5] have attributed decreasing wind power output to anthropogenic afforestation and urbanization that intensifies surface roughness, thus slowing down wind speed. Others[3,6,7] have argued that change in wind speed coincides strongly with variations in various climatic indices of ocean–atmosphere oscillations, thereby proposing that uneven global warming has weakened wind speed by reducing large-scale pressure gradient. A recent published study[8] further pointed out a robust weakening of Hadley circulation, which enables reduced average wind speed and increased extremely low wind speed in tropical and

subtropical regions. Therefore, this decline in Hadley circulation largely provides evidence for drastic change in extreme power shortage events in low- and middle-latitude countries we observed (Fig. 4), which in turn supports the hypothesis that uneven warming and large-scale circulations control the global decline in wind speed.

Change in solar power potential is a result of the combined effects of multiple natural and anthropogenic factors, including solar radiation, ambient temperature, as well as surface wind speed[9–12], among which solar radiation generally rank the most important constraint, although other factors still evince a weak interactive effects (Supplementary Figure 21). Over the past several decades, universal climate warming has strengthened convective updrafts to raise cloud cover that enables reduced solar radiation and increased extremely low solar radiation. Solar radiation is also limited by aerosols that tend to allow decreased solar irradiance received by photovoltaic panels through dimming and soiling effects, thereby undermining solar power potential[13,14]. In addition, the effects of ambient temperature and surface wind speed are largely compounded and mediated via change in operational temperature of photovoltaic modules[2]. Specifically, cool and windy conditions generally improve photovoltaic cell performance through decreasing operational cell temperature, while hot and windless weathers reduce it. Although the impacts of solar radiation, ambient temperature, and surface wind speed sometimes reinforce or offset each other[2], extreme power shortage events of wind-solar mixed system likely exhibit an uptrend in coming future, resulting from foreseeable climate warming that enables weakened solar radiation, increased temperature, and declined wind speed.

Supplementary Note 4. Selection criteria of 42 major countries and the expanded results of the whole 178 countries

As visualizing too many countries may overshadow information about major economies that the scientific community usually pays great attentions, we present some results for 42 major countries (Figs. 4–5). These countries were chosen based on power demand and regional representation. To be specific, we selected the top 10 countries with the highest electricity demand for individual continents (i.e., Asia, Europe, Africa, and America), apart from Oceania in which only two countries (i.e., Australia and New Zealand) were chosen (Supplementary Table 2). The resulting 42 major countries represented ~87% of the total power demand around the world[15]. Our study also shows the corresponding results (Supplementary Figures 16–17) across the global 178 counties (Supplementary Table 1) that covers ~99% of power demand across the globe[15], and the expanded results do not alter our main conclusions.

As shown by Supplementary Figure 16, most of which presented the same trends for the two types of extreme power shortage events across the global 178 countries. Indeed, there are approximately 61% countries where trends in the frequency of extreme long-duration events are in line with those of extreme low-reliability events (upper-right and bottom-left quadrants in Supplementary Figure 16). By contrast, the change in extreme low-reliability events is not

entirely in accordance with that in extreme long-duration events. For example, Geogia exhibited a growth in frequency of extreme long-duration events (+0.80) but a decrease in frequency of extreme low-reliability events (–4.48).

Moreover, changes in extreme power shortage events are unevenly distributed but rather exhibit a latitudinal gradient and an evident disparity in countries with different economic development levels (Supplementary Figure 16). Specifically, developed countries in high latitudes generally show slight variability in extreme power shortage events, yet developing countries in low and middle latitudes tend to have drastic changes in extreme power shortage events. For example, the greatest changes in frequency of extreme power shortage events have emerged in developing countries in low- and middle-latitude countries (e.g., Nepal, Honduras and Costa Rica; blue bubbles at the edge of quadrants in Supplementary Figure 16a); however, high-latitude countries generally exhibit a small variability in frequency of extreme power shortage events.

Supplementary Figure 17 shows climatological drivers underlying the detected growing extreme power shortage events by combining extremely low wind speed and solar radiation across the whole 178 countries. We find that, the growth in extreme shortage events is largely attributable to prolonged extremely low wind speed (change in bubble's colors) and solar radiation (change in bubble's size) during the period between 1980–2000 and 2001–2022. In addition, there are large disproportionalities between changes in extreme power shortage events and variabilities in wind speed and solar radiation. Small changes in annual average wind speed and solar radiation generally indicate considerable changes in compound extremely low wind speed and solar radiation, even resulting in a larger variability in extreme power shortage events. In fact, even though the change in annual average wind speed and solar radiation across the 178 countries is as low as 1.20% (the pink circle in Supplementary Figure 17d), change in annual hours of extremely low wind speed and solar radiation events reached 13.62% (the blue circle in Supplementary Figure 17d–e). Moreover, such 13.62% change in the annual hours of extremely low wind speed results in up to average 21.39% change in in extreme long-duration and low-reliability power shortage events (the red circle in Supplementary Figure 17e).

Supplementary Note 5. Sensitive tests of sensitivity test of wind and solar installed capacities based on the IAMs prediction

Given the potential impact of wind and solar installed capacities on extreme power shortage events and their drivers, we collected wind and solar installed capacities across the world predicted by IAMs in this mid-century from the International Institute for Applied Systems Analysis (IIASA)[16] (https://data.ece.iiasa.ac.at/ar6/). The wind and solar installed capacities for individual countries' have been estimated based upon electricity demand and the global installed capacities predicted by IAMs. We applied the IAMs-based installed capacities of wind

and solar power to examine the variabilities in extreme power shortage events and their driving forces.

Supplementary Figure 24 shows the characteristics of defined extreme long-duration and low-reliability events for wind-solar supply systems during the period 1980–2022 using IAMs-based wind and solar installed capacities. Globally, wind-solar supply systems have experienced an increasing trend in extreme long-duration and low-reliability events, although there are repeated up and down undulations irrespective of their frequency, duration, and intensity. Indeed, long-duration events worldwide increased significantly at a rate of 0.012 per year in frequency (P<0.001; Supplementary Figure 24a), 0.451 hours per year in duration (Supplementary Figure 20b), and 0.181 per year in intensity (Supplementary Figure 24c). In particular, we find that extreme long-duration events over the last two decades considerably outnumbered those over the first two decades. For example, the annual average duration of extreme low-reliability events rose evidently from 26.97 hours during 1980–2000 to 28.61 hours during 2001–2022 (one-way analysis of variance (ANOVA), P<0.001; Supplementary Figure 24e).

We further revealed climatological drivers underlying the detected growing extreme power shortage events by combining extremely low wind speed and solar radiation (Supplementary Figure 25). We find that, the growth in extreme shortage events is largely attributable to prolonged extremely low wind speed (change in colors) and extremely low solar radiation (change in size) during the period between 1980–2000 and 2001–2022. Although variabilities in extreme power shortage events are controlled by both wind and solar drought, their importance differ across different metrics. The impact of wind droughts on the frequency of power shortage events is more significant, while wind drought and solar drought show a nearly identical contribution to the duration and intensity of power shortage events (Supplementary Figure 26).

Supplementary Note 6. Sensitive tests of sensitivity test of wind/solar ratio based on the IAMs prediction

In light of the potential uncertainty of wind/solar proportion towards extreme power shortage events and their driving forces, we acquired the supply share of wind and solar power according to the projections of IAMs by this mid-century from the International Institute for Applied Systems Analysis (IIASA) (https://data.ece.iiasa.ac.at/ar6/; Supplementary Table 6). As shown in Supplementary Table 6, wind power constitutes 39.8–79.6% of wind-solar mix supply systems, with an average of 48.0% across the world.

As shown by Supplementary Figure 27, wind-solar supply systems have experienced an increasing trend in extreme long-duration and low-reliability events, although there are repeated up and down undulations irrespective of their frequency, duration, and intensity. In fact, long-

duration events worldwide increased significantly at a rate of 0.015 per year in frequency ($P<0.001$; Supplementary Figure 27a), 0.426 hours per year in duration (Supplementary Figure 27b), and 0.177 per year in intensity (Supplementary Figure 27c). In particular, we find that extreme long-duration events over the last two decades considerably outnumbered those over the first two decades. For example, the annual average frequency of extreme low-reliability events increased evidently from 35.33 hours during 1980–2000 to 37.98 hours during 2001–2022 (one-way analysis of variance (ANOVA), $P<0.001$; Supplementary Figure 27d).

Supplementary Figure 28 shows that potential climatological drivers underlying the detected growing extreme power shortage events by combining extremely low wind speed and solar radiation. We find that, the growth in extreme shortage events is largely attributable to prolonged extremely low wind speed (change in colors) and extremely low solar radiation (change in size) during the period between 1980–2000 and 2001–2022. Although variabilities in extreme power shortage events are controlled by both wind and solar drought, their importance differ across different extreme events and their metrics (Supplementary Figure 29). For example, the impact of wind droughts on the frequency of long-duration shortage events is more significant (Supplementary Figure 29a), while solar droughts largely dominate the variabilities in the duration and intensity of long-duration shortage events (Supplementary Figure 29b–c).

Supplementary Note 7. Expanded analyses of compound extremely low wind speed and solar radiation events and power shortage events

In order to disentangle the potential driving forces behind the growing power shortage events, we define compound extremely low wind speed and solar radiation events, during which both wind speed and solar radiation are below the 10th percentile of the daily average value across 43 surveyed years. The results showed that the annual hours of defined compound extremely low wind speed and solar radiation during 2001–2022 (500.89 hours on average) was evidently higher than those during 1980–2000 (528.84 hours on average) across the world (Extended Data Fig. 5).

Spatial analyses further illustrated that the observed uptrend in annual hours of defined compound extremely low wind speed and solar radiation is a global-scale phenomenon (Extended Data Fig. 5). There are most countries with growing extreme long-duration events during the period of 1980–2000 and 2001–2022. In fact, there over 70% of surveyed 178 countries with uptrend in annual hours of defined compound extremely low wind speed and solar radiation, although some countries located in North Africa, South America, and Europe evinced a decreasing trend in annual hours of defined compound extremely low wind speed and solar radiation. Moreover, the change in compound extremely low wind speed and solar radiation events also shows a latitude gradient (Extended Data Fig. 5). High-latitude countries (e.g., Canada and Sweden) usually have smaller variabilities in compound extremely low wind

speed and solar radiation, while countries located in low latitudes (such as India and Mozambique) tend to favor larger changes in compound extremely low wind speed and solar radiation.

Supplementary Figure 30 shows that the relationship between changes in extreme power shortage events and variabilities in annual hours of compound extremely low wind speed and solar radiation events. The increases in extreme power shortage events are in accordance with significant growth in annual hours of compound extremely low wind speed and solar radiation events since the 1980s. For instance, highly frequent extreme power shortage events in the recent period (e.g., 2022, 2016, 2010, and 2013) have resulted from comparatively long annual hours of compound extremely low wind speed or solar radiation events, while a lower frequency of extreme power shortage events before 2000 (e.g., 1983 and 1994) is ascribed to shorter periods of compound extremely low wind speed or solar radiation events.

Moreover, we have compared the change in average wind speed and solar radiation, compound extremely low wind speed and solar radiation events, as well as extreme power shortage events during the period of 1980–2000 and 2001–2022 (Figure 5d–e). The results showed that a comparatively small change in average wind speed and solar radiation usually suggest a large variability in compound extremely low wind speed and solar radiation event. Indeed, the change in average wind speed and average solar radiation of 1.0% (the pink circle in Fig. 5d) imply 12.5% variability in annual hours compound extremely low wind speed and solar radiation events (the blue circle in Fig. 5d–e), which further triggers off over 30% change in extreme power shortage events (the red circle in Fig. 5e).

Supplementary Note 8. Expanded discussion on temporal resolution of wind speed data

Although this study that evaluates the reliability of wind-solar power supply systems based upon an hourly climatologic reanalysis data can capture the variability characteristics of potential power shortage events, wind speed varies dramatically even on a minute-scale. Therefore, it is necessary to perform a higher temporal resolution of analyses. However, it is unfortunate that minute-scale wind speed and power demand data remain unavailable, which limited our modelling and simulation of extreme power shortage events at a higher temporal resolution analysis. Thus, minute-scale meteorology data should support follow-up refined analysis on extreme power shortage events, if minute-scale data become accessible in the future.

Supplementary Note 9. Sensitive tests of system reliability and power shortage events with consideration of single-axis tracking system and solar tracking technology progress

Our study based upon dual-axis tracking system may result in potential uncertainty of system reliability and power shortage events. Therefore, we have added new simulations of single-axis tracking type during the period of 1980–2000. It shows that the reliability of wind-solar supply systems would be slightly underestimated across the world, with the change in system reliability

ranging from –0.008 to 0.002 (Supplementary Figure 32). We further examined the impact of solar tracking types on extreme power shortage events of wind-solar supply systems during the period through 1980–2000. The results based upon single-axis tracking system result likely in an underestimate of extreme events in middle- and low-latitude countries but an overestimate of extreme events in high-latitude countries (Supplementary Figure 33). Additionally, we investigate the influence of tracking system progress on the trend in extreme power shortage events since 1980, during which we assume one-axis solar tracking system over the period of 1980–2000 but dura-axis solar tracking system between 2001 and 2022. We find, that solar tracking type progress does not alter the observed increasing trends in both long-duration and low reliability events of wind-solar electricity system since 1980s (Supplementary Figures 34–36).

Supplementary Note 10. Sensitive tests of power shortage events with consideration of concentrating solar power technology (CSP)

Our study mainly involves the impact of climate change on the reliability of wind-solar supply systems through excluding flexible electricity resources such as energy storage, and thus CSP technology is not in consideration for the solar supply. However, given potential importance of CSP technology in the reliability of solar supply system, we have added a sensitivity test that integrates CSP and photovoltaic solar technologies based on their future supply shares by the mid-century. The future shares of CSP and photovoltaic solar technologies are obtained from the International Institute for Applied Systems Analysis (IIASA) (https://data.ece.iiasa.ac.at/ar6/; Supplementary Table 5). We calculated CSP supply output potential according to solar direct irradiance and ambient temperature[9,17].

The results with consideration of CSP technologies do not alter our main conclusions (see Supplementary Figures 37–41). As shown in Supplementary Figures 37–38, our estimates combined with CSP technology also shows an uptrend trend in long-duration extreme power shortage events since 1980, and such an increasing trend is of global significance. We also examined the change in low-reliability events during the period of 1980–2022, and the results can also capture the increasing low-reliability events across the world, despite some different estimates in Middle East compared to our initial results (see Figure 3, Supplementary Figures 39–40). Besides, we further investigate the relationship between changes in extreme events and variabilities in climatic variables, which still supports our finding that uptrend in compound extremely low wind speed and solar radiation events is behind the growing extreme power shortage events (Supplementary Figure 41).

Supplementary Note 11. Sensitive tests of economics and renewable policy through using the wind-solar share predicted by REMIND model

Our study estimates the install capacities of wind and solar power using the IEA predicted

wind-solar share that have considered driving factors including economics, geographics, and renewable policies. However, as the potential importance and uncertainty of economics and renewable policies towards the wind-solar share of power generation, we have further performed a series of sensitivity tests based upon REMIND model[17,18]. REMIND is a well-known integrated assessment model (IAM) that coupled macroeconomic growth and renewable polices into the assessment of future electricity supply mix (i.e., wind and solar). Here, we employed the wind-solar share in 2050 under 1.5°C climate target projected by the REMIND model (Supplementary Table 4), to calculate the wind-solar installed capacities and the extreme power shortage events. As shown by Supplementary Figures 44–48, the results also underpin growing extreme power shortage events of wind-solar electricity system across the world, albeit with downtrends in a few countries such as Sudan and Saudi Arabia (see Supplementary Figures 44–47). Furthermore, we explored the potential mechanisms underlying such phenomenon, which also highlights compound extremely low wind speed and solar radiation events may be responsible for the observed growing extreme power shortage events (see Supplementary Figure 48).

Supplementary Note 12. The method to estimate the optimal generation ratio of wind to solar

We determined the optimal generation ratio of wind to solar for each country and region under the highest reliability of power system. Firstly, we generated a set of wind-solar generation mixes, ranging from 0 to 100% at an interval of 1% (e.g., 1% solar and 99% wind, 10% solar and 90% wind, 90% solar and 10% wind, 1% solar and 99% wind). Then, these wind-solar mixes and hourly electricity demand data, together with area-weighted average hourly solar and wind capacity factors, were applied to compute average reliability of wind-solar power system. Finally, we determined the wind-solar mix with the highest average reliability as the optimal generation ratio of wind to solar for individual countries (Supplementary Figure 50).

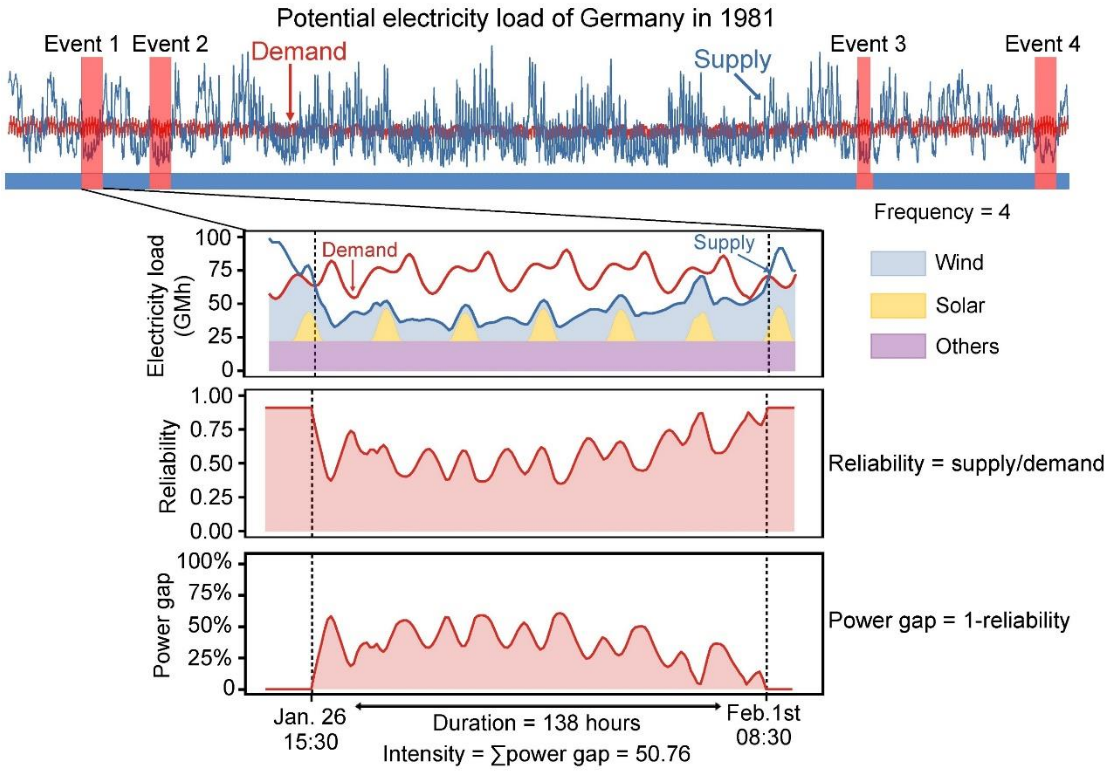

Supplementary Figure 1 | Schematic diagram of frequency, duration, and intensity of extreme power shortage events. There were four extreme long-duration events (frequency=4) in Germany in 1981, and one of these events occurred during the period from 15:30 on Jan. 26 to 08:30 on Feb. 1st (duration=138 hours), with the total power gap of 50.76 (intensity=50.76).

	Long-duration events						Low-reliability events					
	Frequency		Duration		Intenisty		Frequency		Duration		Intenisty	
	MERRA-2	ERA5	MERRA-2	ERA5	MERRA-2	ERA5	MERRA-2	ERA5	MERRA-2	ERA5	MERRA-2	ERA5
**Annual mean**	4.8	4.4	151.1	144.8	51.1	48.9	22.0	19.1	59.9	53.0	20.5	18.2
**1980**	4.6	4.1	147.3	145.2	49.4	48.5	23.2	19.9	51.2	41.9	17.8	14.6
**1981**	4.4	4.0	144.9	134.0	48.2	44.3	23.0	20.2	58.3	59.0	20.1	20.1
**1982**	4.7	4.3	155.7	148.4	52.4	50.0	22.7	20.2	62.0	57.4	21.2	19.5
**1983**	4.1	3.7	141.8	134.2	46.7	44.0	21.3	18.2	61.0	52.2	20.6	17.7
**1984**	4.5	4.0	144.1	140.4	48.4	47.4	22.8	20.4	57.3	53.1	19.5	18.1
**1985**	4.7	4.4	150.1	148.8	50.6	50.7	22.2	19.2	51.7	59.8	17.9	20.1
**1986**	4.3	3.9	140.3	136.6	47.9	46.9	21.0	18.0	51.0	47.9	17.6	16.5
**1987**	5.0	4.4	146.0	137.2	48.9	45.9	22.5	19.0	53.1	45.3	18.3	15.7
**1988**	4.4	4.0	146.3	142.2	49.0	47.8	20.3	18.1	51.8	47.5	18.0	16.4
**1989**	4.4	4.0	151.6	147.8	51.8	50.2	21.8	19.1	57.0	48.5	19.6	16.8
**1990**	4.3	3.9	150.0	141.6	50.4	47.8	20.4	17.5	63.7	54.4	21.7	18.7
**1991**	4.7	4.4	151.6	144.4	52.0	49.7	21.2	18.1	58.6	49.7	20.1	17.1
**1992**	4.6	4.2	142.1	134.8	47.7	45.4	20.7	17.5	51.9	45.5	17.9	15.7
**1993**	4.3	3.8	142.2	138.1	47.2	46.3	20.3	17.3	51.5	48.5	17.5	16.5
**1994**	4.3	4.1	139.8	128.9	46.5	43.4	20.7	18.0	55.9	49.9	19.2	17.0
**1995**	4.8	4.4	141.3	137.7	47.0	46.2	21.3	18.7	58.6	48.6	20.0	16.7
**1996**	4.8	4.3	147.2	141.4	49.6	48.1	21.5	18.7	49.6	47.2	17.3	16.3
**1997**	4.8	4.3	151.6	144.8	52.1	50.0	21.1	18.4	62.6	60.9	21.5	20.7
**1998**	4.6	4.2	146.9	145.9	49.8	49.2	19.6	17.9	63.6	58.4	21.7	19.8
**1999**	4.6	4.3	153.8	146.9	51.2	49.0	21.4	18.6	63.5	55.1	21.7	19.0
**2000**	4.6	4.1	151.2	140.9	51.3	47.4	21.0	18.1	50.7	44.3	17.6	15.4
**2001**	4.6	4.1	149.0	140.8	50.0	47.3	20.5	17.3	46.7	44.5	16.2	15.4
**2002**	4.3	3.8	143.1	140.9	48.6	47.9	20.4	17.8	48.1	44.5	16.8	15.5
**2003**	4.9	4.3	153.5	144.3	51.8	49.0	20.9	18.4	54.9	51.6	18.9	17.7
**2004**	4.5	4.1	150.9	149.2	51.5	51.5	21.1	18.7	59.0	55.4	20.2	18.8
**2005**	5.2	4.8	151.0	138.4	51.2	46.7	22.2	19.6	55.4	47.2	19.1	16.3
**2006**	5.3	5.0	159.3	152.8	54.3	52.2	22.0	19.7	60.5	50.8	20.8	17.6
**2007**	4.7	4.3	154.3	143.3	51.9	48.1	21.1	18.8	67.7	63.6	23.3	21.8
**2008**	5.4	5.1	163.7	156.3	55.7	53.5	22.6	20.2	62.3	54.0	21.5	18.7
**2009**	5.0	4.7	157.1	153.4	53.0	51.5	22.1	20.1	59.5	53.5	20.5	18.3
**2010**	5.7	5.3	154.6	147.5	53.0	50.4	24.4	21.6	69.3	60.4	23.8	20.7
**2011**	5.8	5.3	162.1	154.3	55.2	52.2	22.5	20.0	69.1	58.2	23.6	19.9
**2012**	5.3	4.8	152.6	147.6	51.2	49.9	23.4	20.1	59.2	63.0	20.6	21.5
**2013**	5.3	4.7	161.5	160.6	54.3	53.8	21.9	19.4	78.6	54.7	26.5	18.9
**2014**	5.3	4.7	154.9	148.5	52.6	50.0	23.2	20.9	64.3	57.2	22.0	19.5
**2015**	5.0	4.5	159.8	148.6	54.6	50.9	22.1	19.4	65.2	53.8	22.4	18.7
**2016**	5.5	5.0	153.1	145.1	52.2	49.2	23.5	21.2	74.2	55.6	24.9	19.0
**2017**	5.3	4.9	153.5	144.7	52.3	48.6	23.7	20.6	63.1	56.5	21.9	19.4
**2018**	4.8	4.3	159.3	147.8	53.7	49.8	23.6	21.0	65.2	64.3	22.4	21.9
**2019**	5.2	4.6	151.9	145.9	51.0	48.7	23.3	20.5	54.9	49.2	19.0	17.1
**2020**	5.3	4.7	155.0	154.9	53.7	53.9	22.8	19.5	74.3	55.2	25.2	19.1
**2021**	5.2	4.4	154.1	146.0	51.1	47.6	23.4	18.4	67.6	58.1	23.2	19.7
**2022**	5.5	4.8	157.7	155.7	54.2	53.0	23.4	18.8	70.4	51.0	24.1	17.7

Supplementary Figure 2 | Comparison of interannual variability in extreme power shortage events between MERRA-2 and ERA5 data. Shading in each block represents annual average power shortage events during the period of 1980–2022, with the colour range from red (high values) to bule (low values).

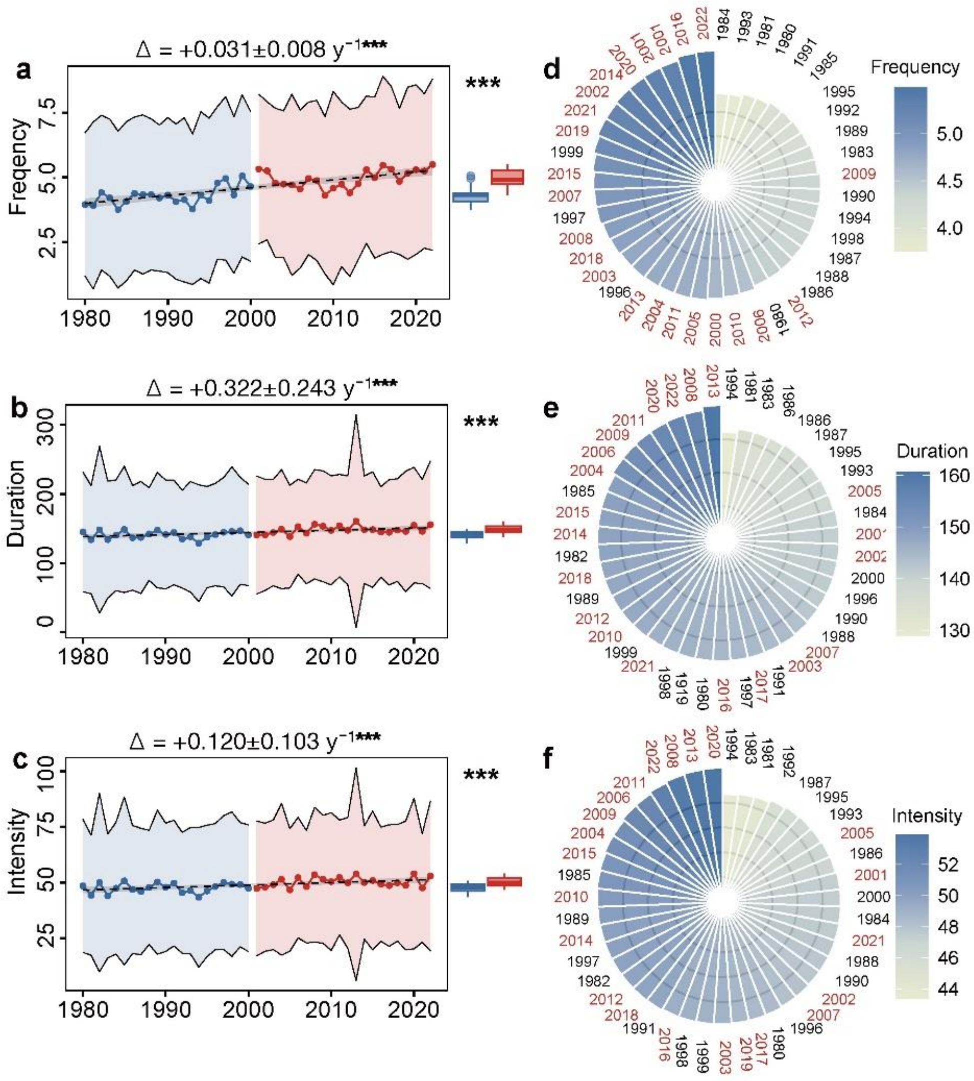

Supplementary Figure 3 | Interannual variability in extreme long-duration events since 1980s using ERA5 data. a-c, Interannual changes in the frequency **(a)**, duration **(b)**, and intensity **(c)** of extreme long-duration events since 1980. The coefficients at the top of the panels indicate robust Theil-Sen's slopes and their corresponding *P* values, examined by Mann-Kendall (MK) test. The right boxplots denote the difference before 2000 (blue) and after 2000 (red) examined by analysis of variance (ANOVA). *** represents the significance under the level of P<0.001. Black dashed lines denote linear fitting of annual average values across surveyed 178 countries. **d-f**, Rank ordering of the annual average frequency **(d)**, duration **(e)**, and intensity **(f)** of extreme long-duration events across the surveyed 43 years, in which years between 1980 and 2000 are labelled with black and those between 2001 and 2022 are labelled with red.

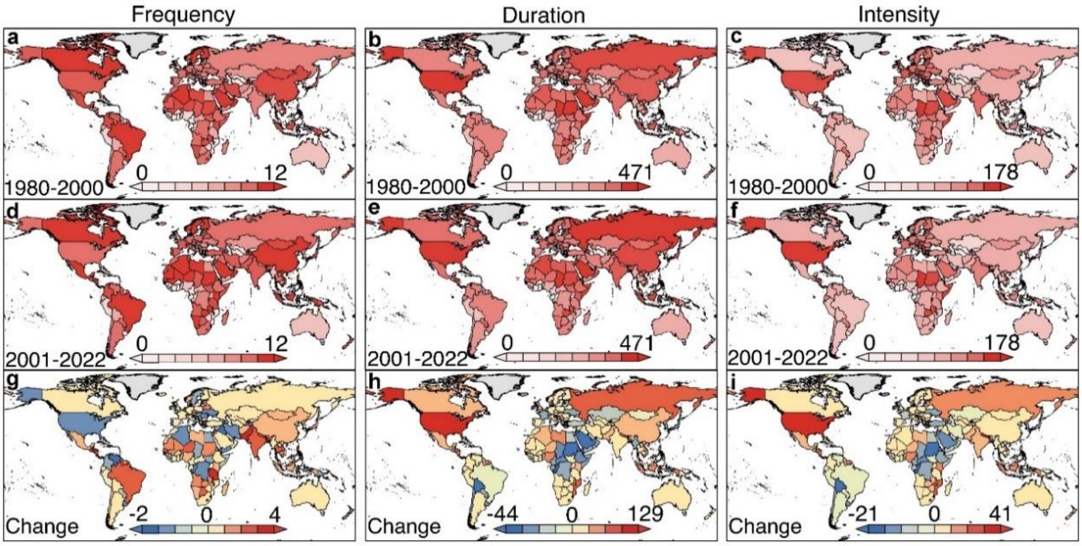

Supplementary Figure 4 | Maps of extreme long-duration events during the period 1980–2000 and 2001–2022 using ERA5 data. a-c, Annual average frequency **(a)**, duration **(b)**, and intensity **(c)** of extreme long-duration events during the period 1980–2000. **d-f**, Annual average frequency **(d)**, duration **(e)**, and intensity **(f)** of extreme long-duration events during the period 2001–2022. **g-i**, Changes in annual average frequency **(g)**, duration **(h)**, and intensity **(i)** of extreme long-duration events between 1980–2000 and 2001–2022.

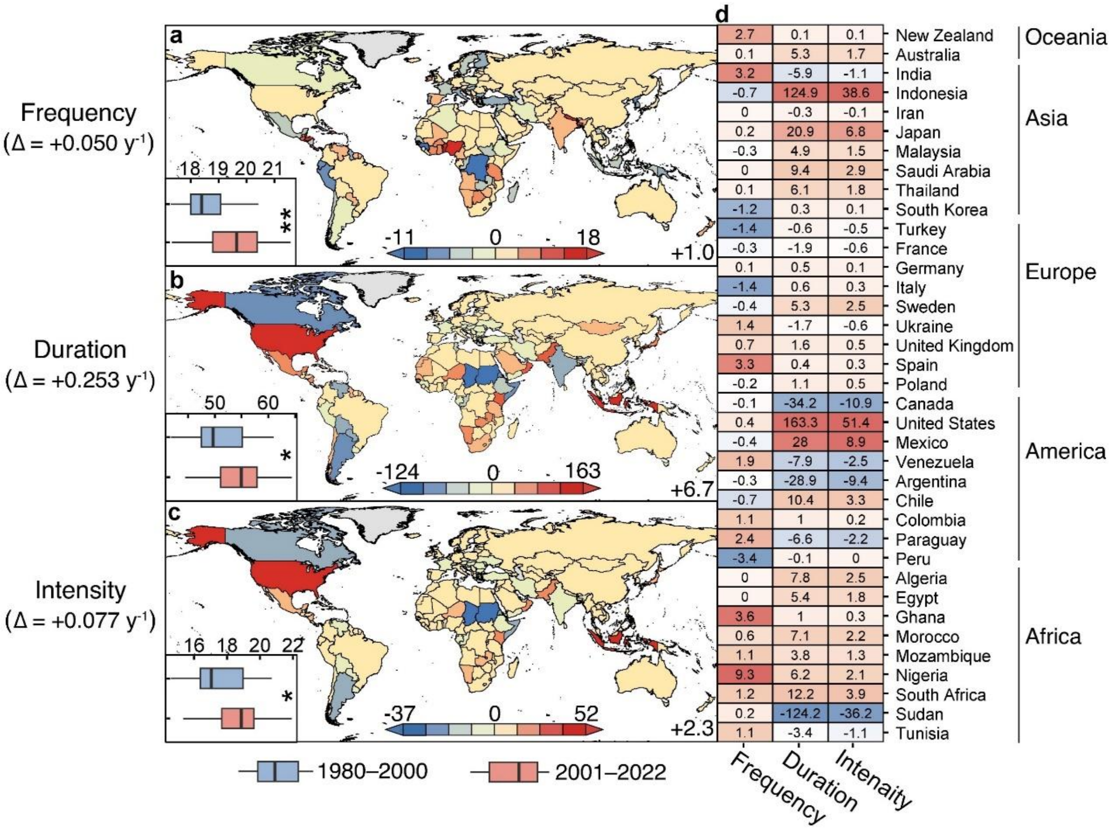

Supplementary Figure 5 | Changes in extreme low-reliability events since 1980 using ERA5 data. **a-c**, Changes in annual average frequency **(a)**, duration **(b)**, and intensity **(c)** of extreme low-reliability events between 1980–2000 and 2001–2022. The left-bottom boxplots denote the difference between 1980–2000 (blue) and 2000–2022 (red) examined by ANOVA, where * and ** represent the significance under the level of $P<0.05$ and $P<0.01$, respectively. **d**, Changes in extreme low-reliability events for 42 major countries between 1980–2000 and 2001–2022.

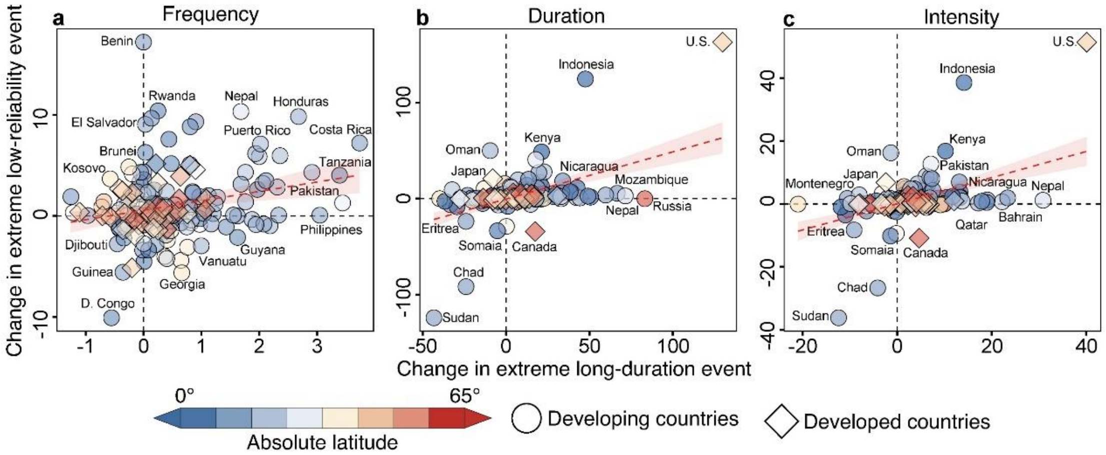

Supplementary Figure 6 | Relationship of changes in extreme long-duration and low-reliability events at the national scale calculated through ERA5 data. Changes in frequency **(a)**, duration **(b)**, and intensity **(c)** of extreme long-duration and low-reliability events for 178 surveyed countries between 1980–2000 and 2001–2022. Red digits represent the proportion of countries that fall in the quadrant. Dashed red lines with shallow red shading are linear fittings between the two types of events and their 95% confidence intervals. D. Congo denotes Democratic Republic of the Congo.

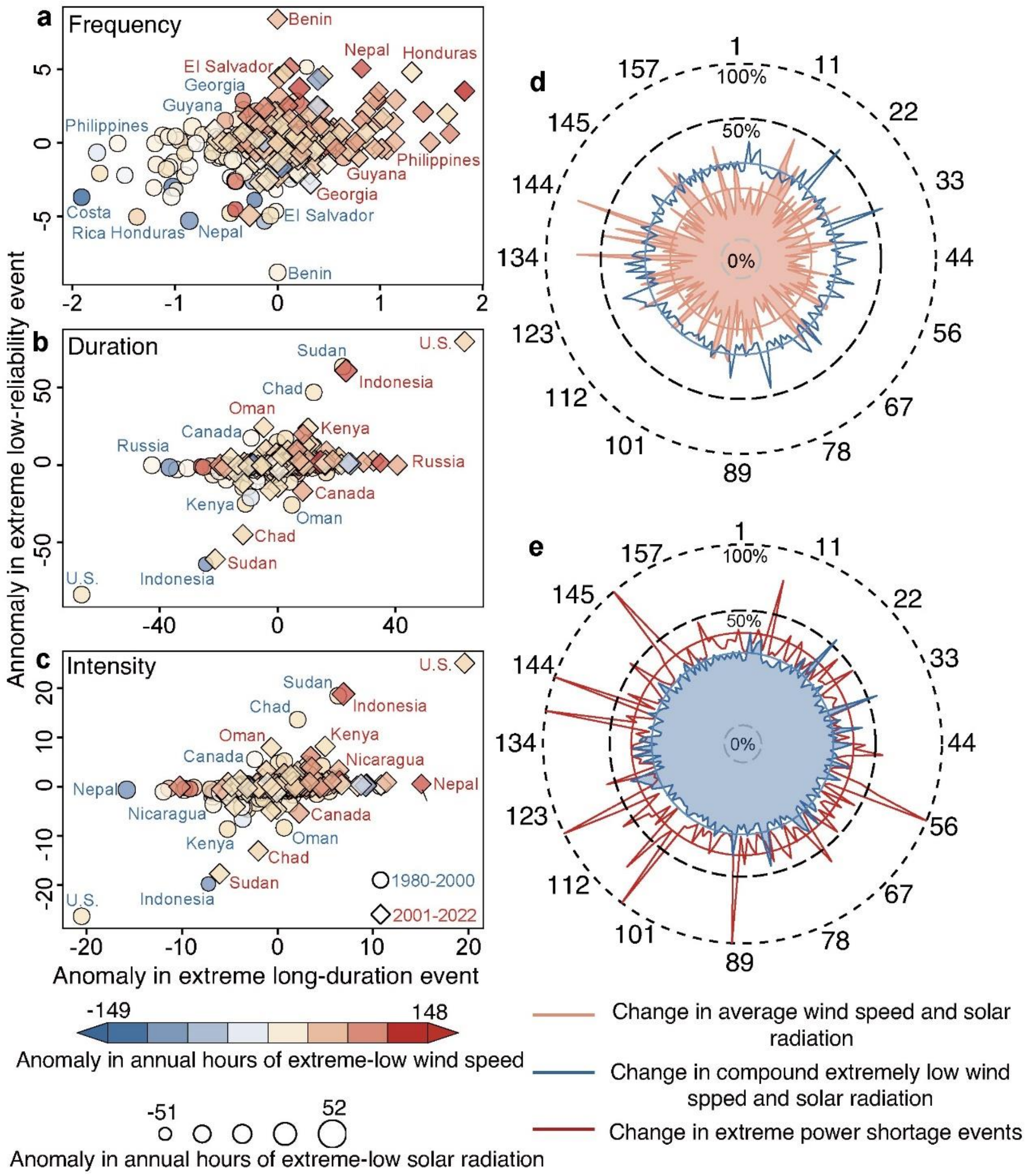

Supplementary Figure 7 | Changes in extreme power shortage events with extremely low wind speed and solar radiation using ERA5 data. a-c, Relationship between anomalies in frequency **(a)**, duration **(b)**, and intensity **(c)** of extreme power shortage events and anomalies in annual hours of extremely low solar radiation and wind speed at hub height for the 178 surveyed countries. **d**, Relationship between change in annual average wind speed and change in annual hours of compound extremely low wind speed & solar radiation between 1980–2000 and 2001–2022. **e**, Relationship between the relative change in annual hours of extremely low wind speed & solar radiation and the relative change in extreme power shortage events between 1980–2000 and 2001–2022. The relative change above 100% is visualized as 100% change. The lines and circles denote individual country's change and the average changes across 178 surveyed countries, respectively. The surrounding digits represent the order number of the 178 countries that are listed in Supplementary Table 7.

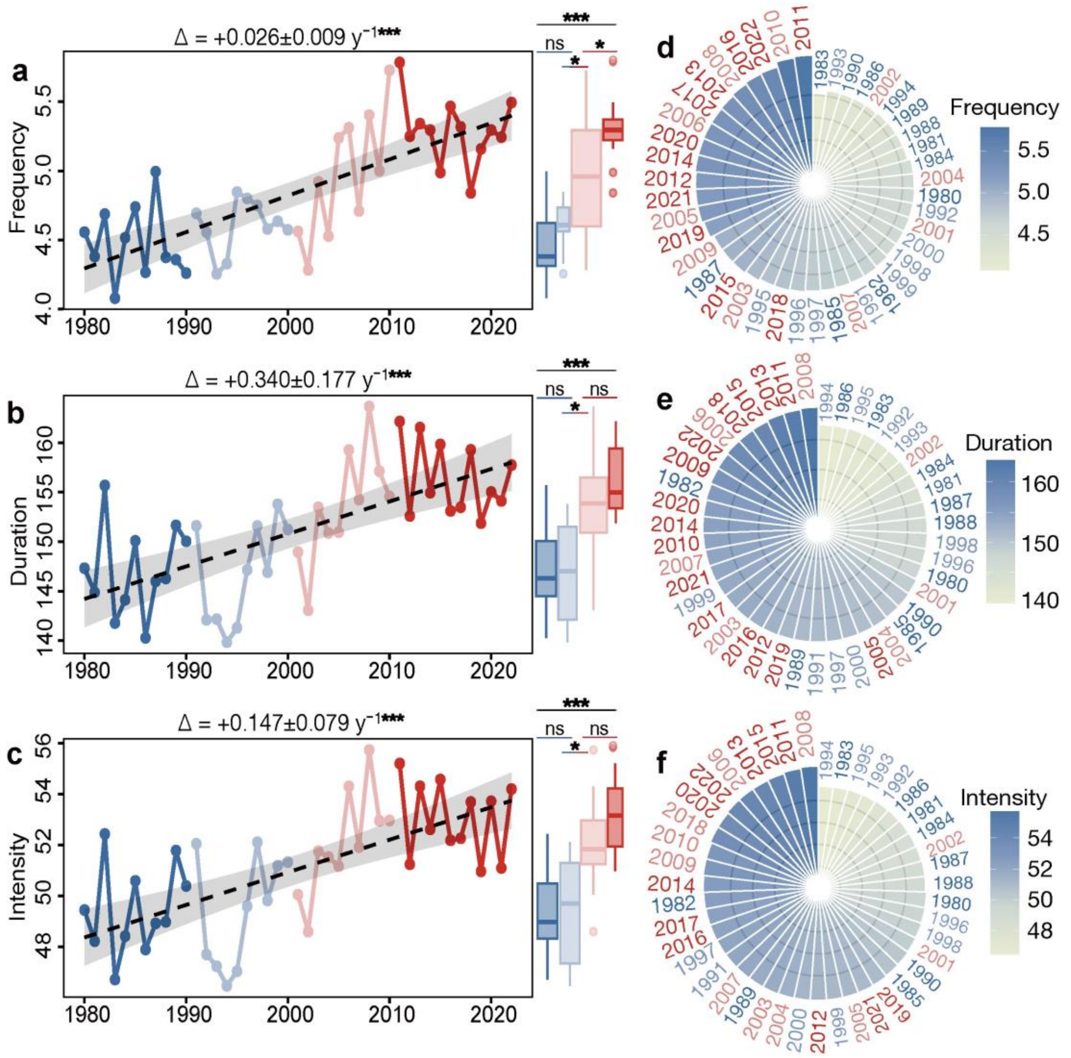

Supplementary Figure 8 | Interannual variability in extreme long-duration events over the past 4 decades. a-c, Interannual changes in the frequency **(a)**, duration **(b)**, and intensity **(c)** of extreme long-duration events since 1980. The coefficients at the top of the panels indicate robust Theil-Sen's slopes and their corresponding *P* values, examined by Mann-Kendall (MK) test. *** represents the significance under the level of P<0.001. The right boxplots denote the difference among the periods of 1980–1990, 1991–2000, 2001–2010, and 2011–2022. Black dashed lines denote linear fitting of annual average values across 178 countries. **d-f**, Rank ordering of the annual average frequency **(d)**, duration **(e)**, and intensity **(f)** of extreme long-duration events across the surveyed 43 years, for which the periods of 1980–1990, 1991–2000, 2001–2010, and 2011–2022 are coloured with deep blue, shallow blue, shallow red, and deep red, respectively.

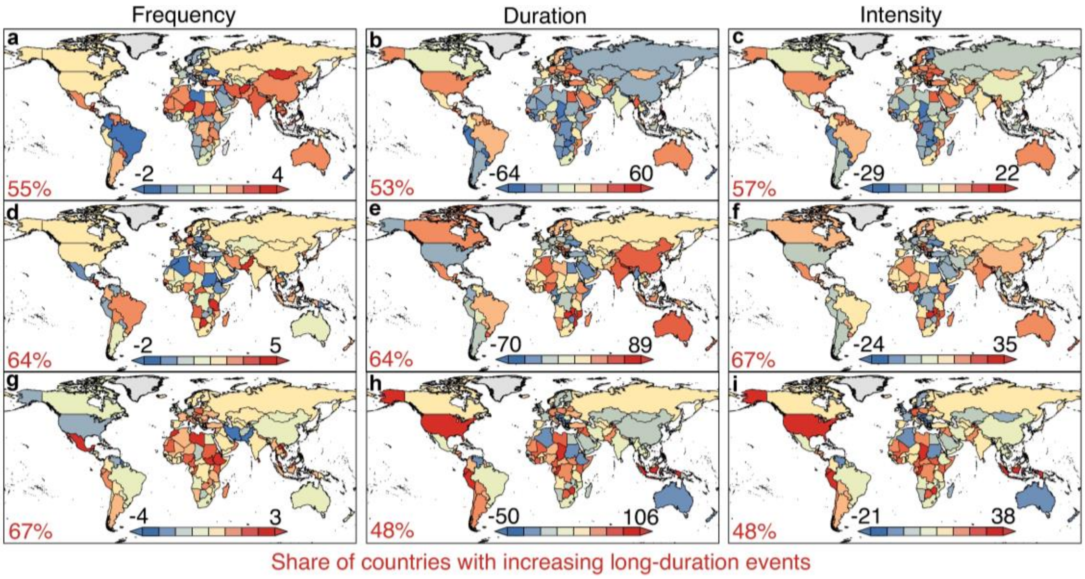

Supplementary Figure 9 | Changes in extreme long-duration events over the past 4 decades. a-c, Changes in annual average frequency **(a)**, duration **(b)**, and intensity **(c)** of extreme long-duration events during the period 1980–1990 and 1991–2000. **d-f**, Changes in annual average frequency **(d)**, duration **(e)**, and intensity **(f)** of extreme long-duration events during the period 1991–2000 and 2001–2010. **g-i**, Changes in annual average frequency **(g)**, duration **(h)**, and intensity **(i)** of extreme long-duration events between 2001–2010 and 2011–2022. The right digits denote the share of countries with increasing long-duration events.

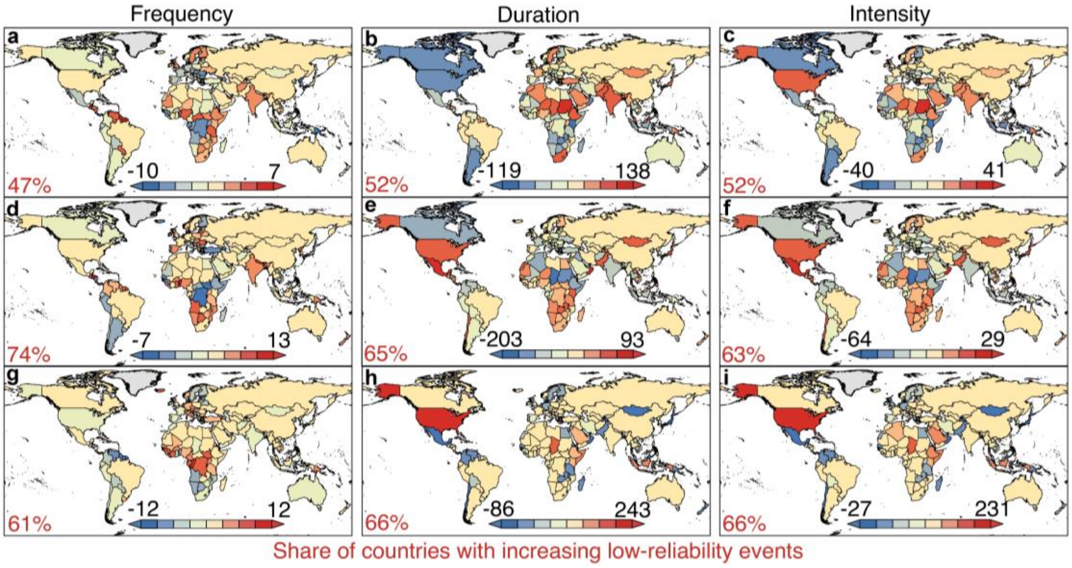

Supplementary Figure 10 | Changes in extreme low-reliability events over the past 4 decades. a-c, Changes in annual average frequency **(a)**, duration **(b)**, and intensity **(c)** of extreme low-reliability events during the period 1980–1990 and 1991–2000. **d-f**, Changes in annual average frequency **(d)**, duration **(e)**, and intensity **(f)** of extreme low-reliability events during the period 1991–2000 and 2001–2010. **g-i**, Changes in annual average frequency **(g)**, duration **(h)**, and intensity **(i)** of extreme low-reliability events between 2001–2010 and 2011–2022. The right digits denote the share of countries with increasing low-reliability events.

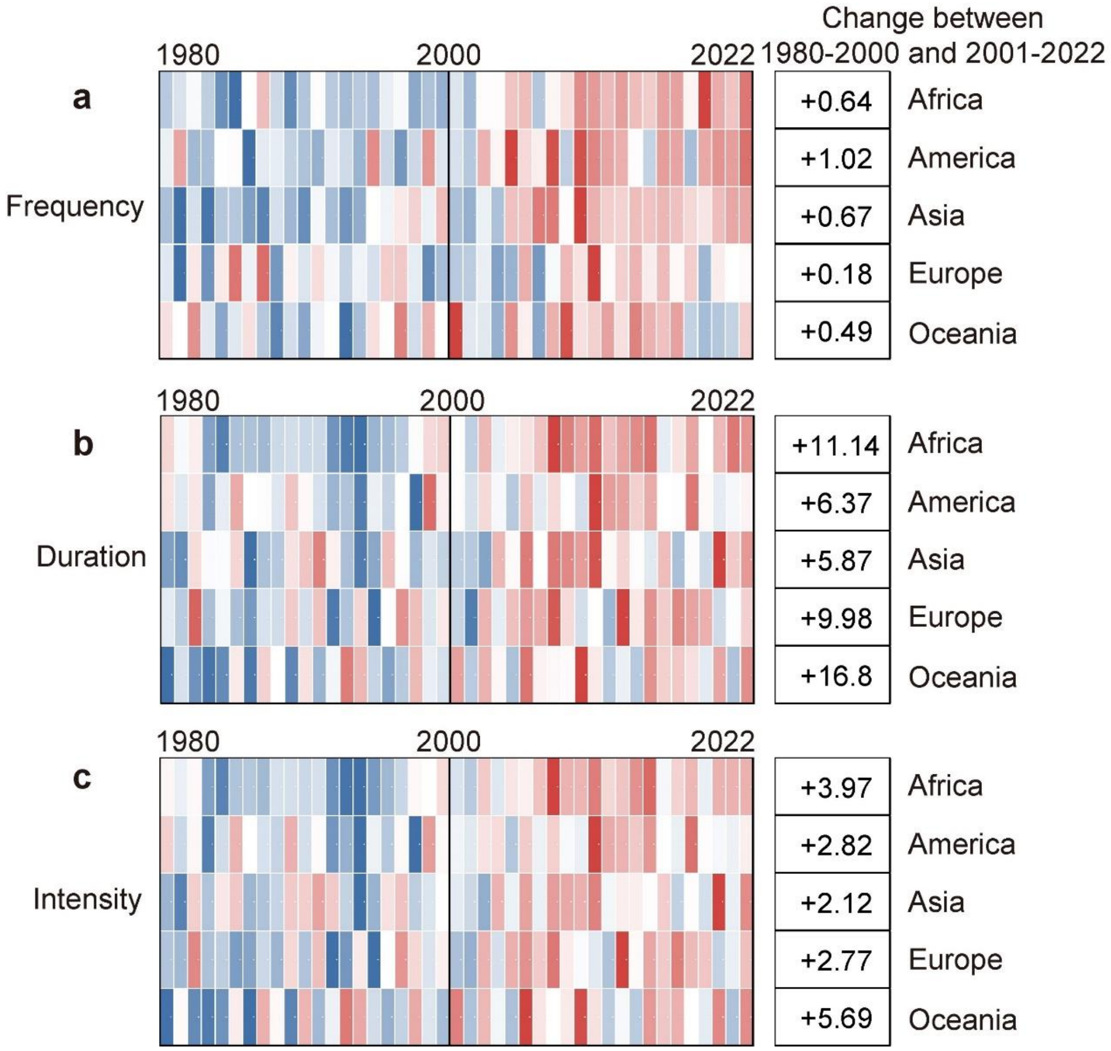

Supplementary Figure 11 | Interannual variability in extreme long-duration events across different continents for the selected 178 countries. a-c, Annual average frequency **(a)**, duration **(b)**, and intensity **(c)** and their changes in extreme long-duration events at the continental scale between 1980–2000 and 2001–2022. The right column summarizes the two-decadal changes in extreme long-duration events for individual continents between 1980–2000 and 2001–2022.

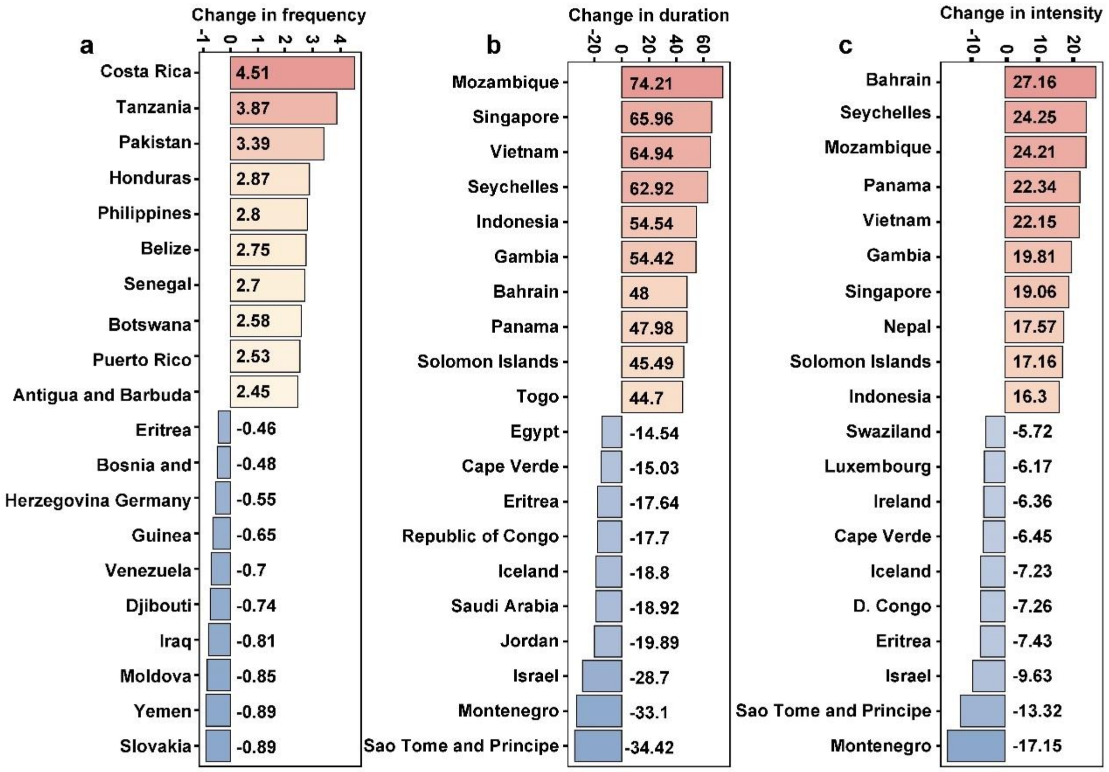

Supplementary Figure 12 | Top 10 countries with the largest increase and decrease in extreme long-duration events for the survey 178 countries between 1980–2000 and 2001–2022. Top 10 countries with the largest increase and decrease in frequency **(a)**, duration **(b)**, and intensity **(c)** of extreme long-duration events across the world between 1980–2000 and 2001–2022.

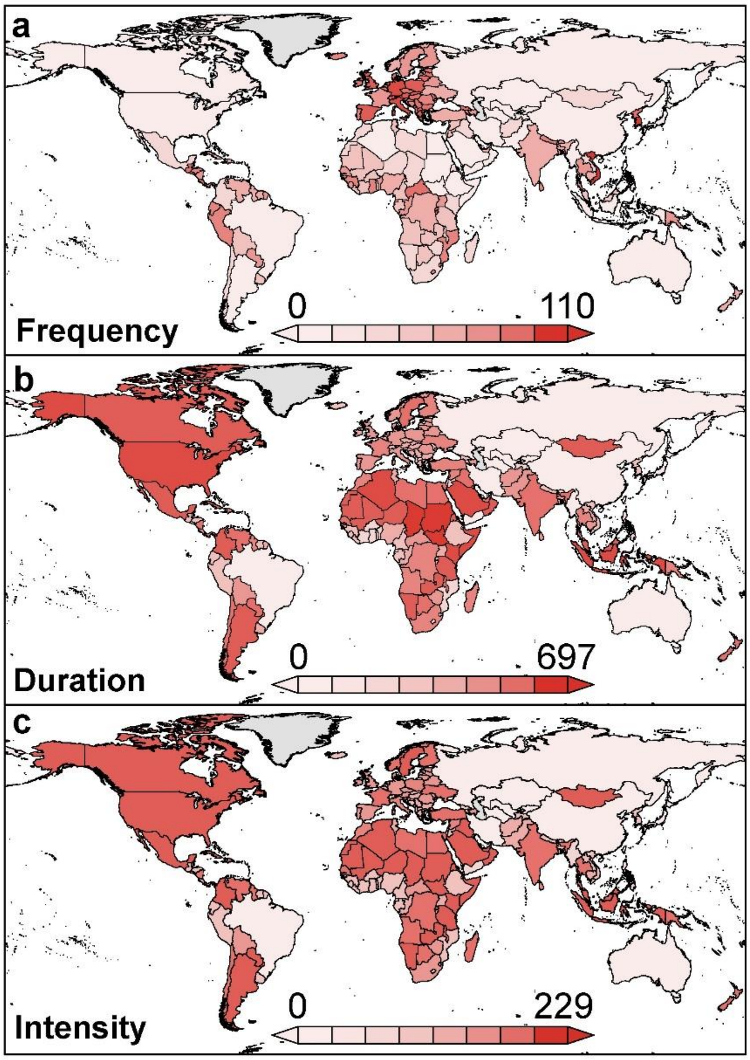

Supplementary Figure 13 | Annual average extreme low-reliability events across the surveyed 178 countries between 1980–2000 and 2001–2022. a-c, Annual average frequency **(a)**, duration **(b)**, and intensity **(c)** of extreme low-reliability events between 1980-2000 and 2001-2022.

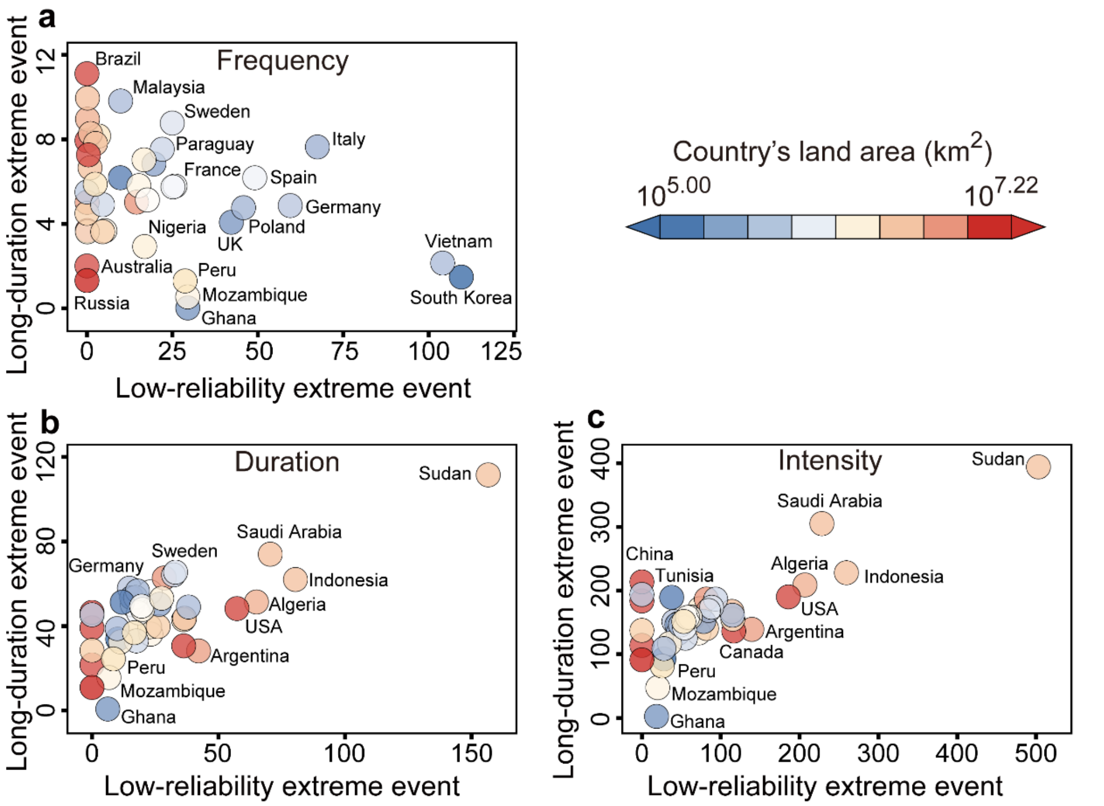

Supplementary Figure 14 | Relationship between extreme low-reliability events and extreme long-duration events of the 42 major countries between 1980–2000 and 2001–2022. a-c, Relationship in frequency **(a)**, duration **(b)**, and intensity **(c)** of extreme low-reliability and long-duration events between 1980-2000 and 2001-2022. Each bubble is colored from red to blue according to their land areas.

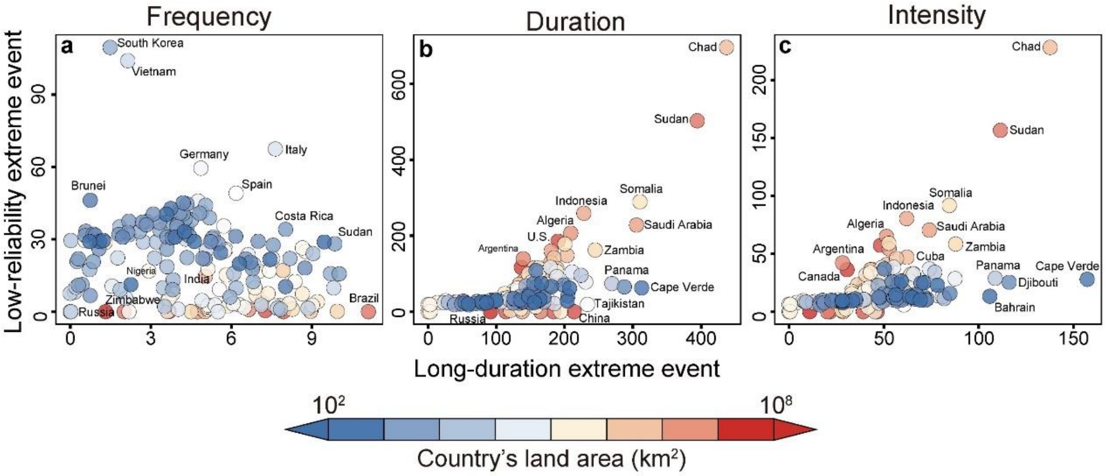

Supplementary Figure 15 | Relationship between extreme low-reliability events and extreme long-duration events across the surveyed 178 countries. a-c, Relationship in frequency **(a)**, duration **(b)**, and intensity **(c)** of extreme low-reliability and long-duration events. Each circle is colored from red to blue according to their land areas.

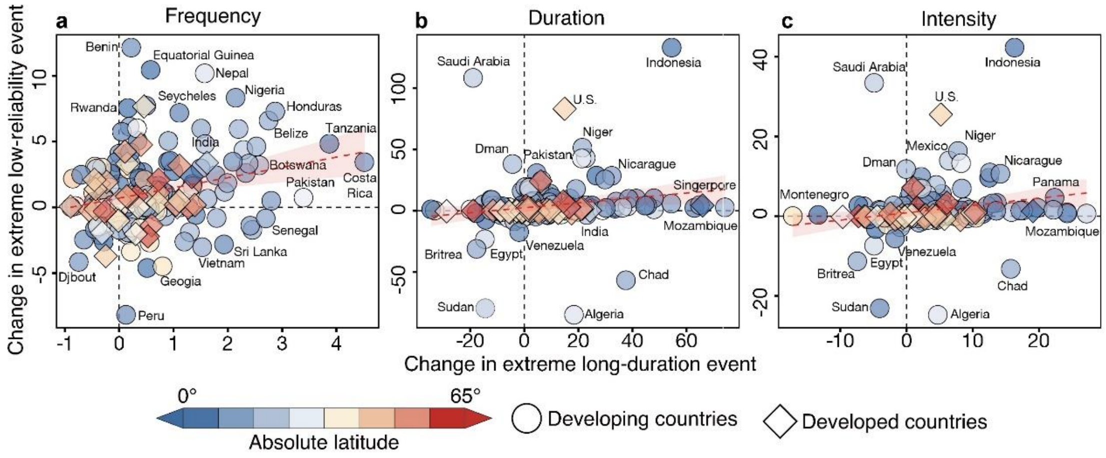

Supplementary Figure 16 | Relationship of changes in extreme long-duration and low-reliability events for the selected 178 countries. Changes in frequency **(a)**, duration **(b)**, and intensity **(c)** of extreme long-duration and low-reliability events for 178 surveyed countries between 1980–2000 and 2001–2022. Red digits represent the proportion of countries that fall in the quadrant. Dashed red lines with shallow red shading are linear fittings between the two types of events and their 95% confidence intervals.

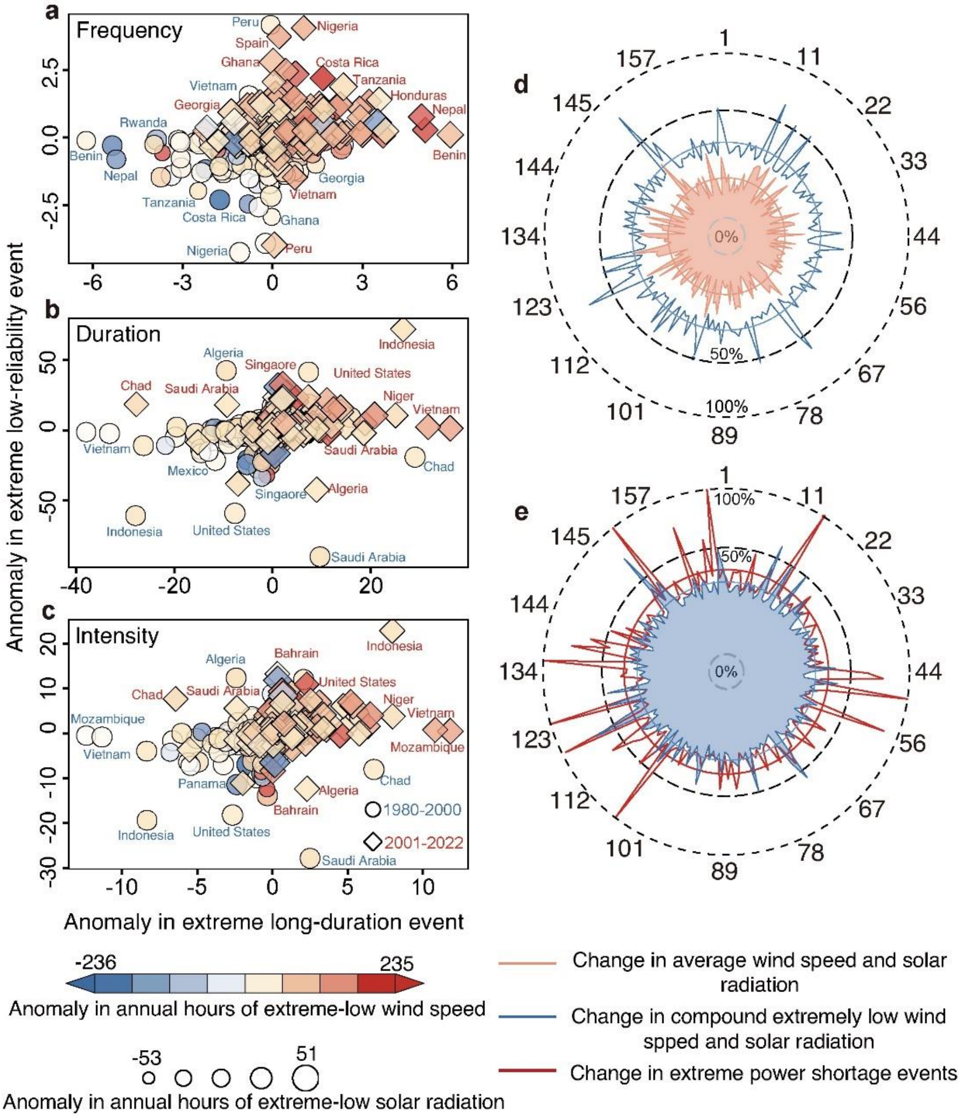

Supplementary Figure 17 | Changes in extreme power shortage events with extremely low wind speed and solar radiation for the selected 178 countries. a-c, Relationship between anomalies in frequency **(a)**, duration **(b)**, and intensity **(c)** of extreme power shortage events and anomalies in annual hours of extremely low solar radiation and wind speed at hub height for the 178 surveyed countries. **d**, Relationship between change in annual average wind speed and change in annual hours of compound extremely low wind speed & solar radiation between 1980–2000 and 2001–2022. **e**, Relationship between the relative change in annual hours of extremely low wind speed & solar radiation and the relative change in extreme power shortage events between 1980–2000 and 2001–2022. The relative change above 100% is visualized as 100% change. The lines and circles denote individual country's change and the average changes across 178 surveyed countries, respectively. The surrounding digits represent the order number of the 178 countries that are listed in Supplementary Table 7.

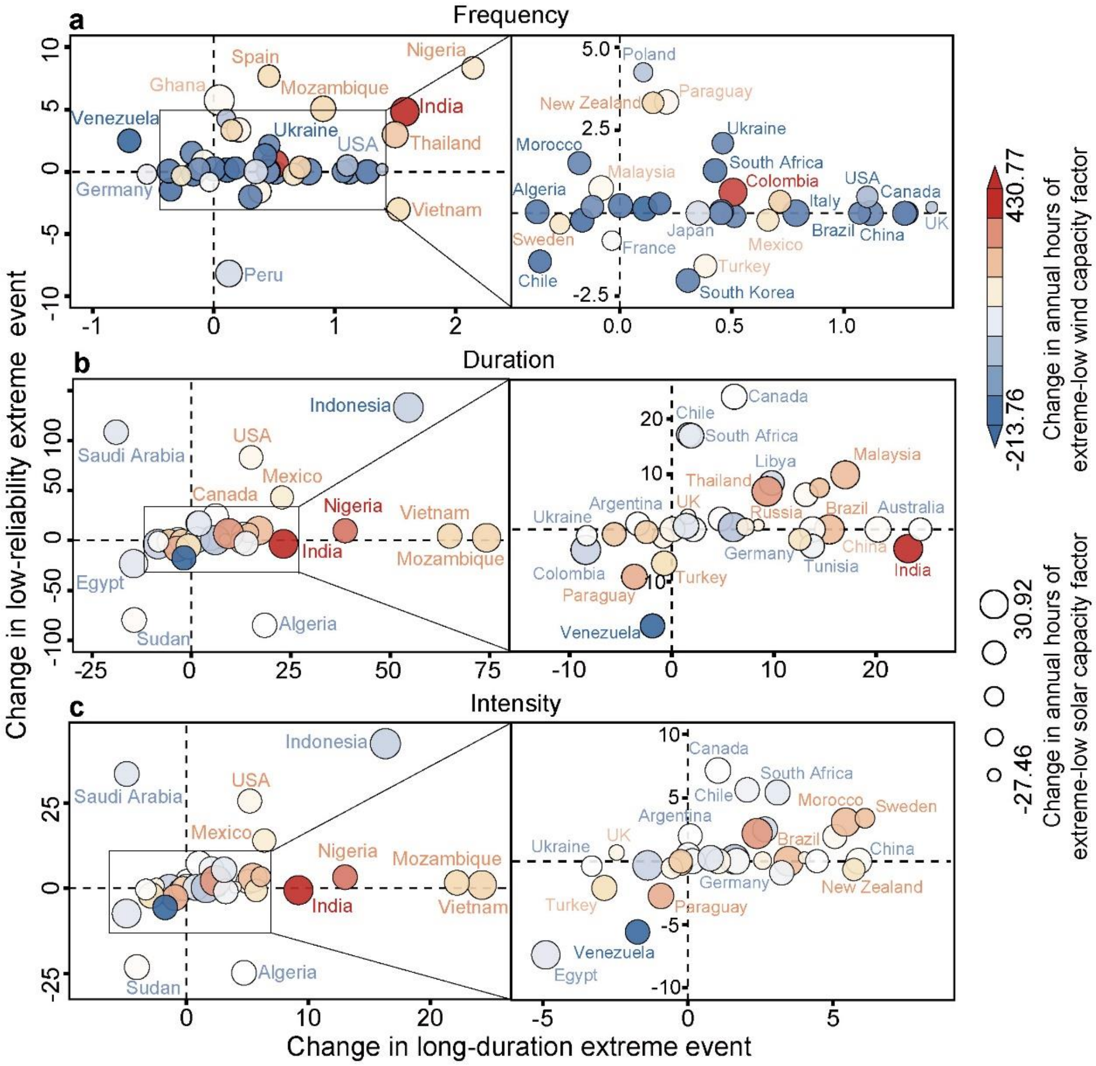

Supplementary Figure 18 | Relationship between changes in extreme power shortage events and trends in extremely low wind and solar capacity factors. a-c, Changes in frequency **(a)**, duration **(b)**, and intensity **(c)** of extreme power shortage events with annual hours of extremely low solar and wind capacity factors for 42 major countries between 1980–2000 and 2001–2022. Extremely low solar and wind capacity factors are defined as below the 10th percentile of daily average value across the surveyed 43 years.

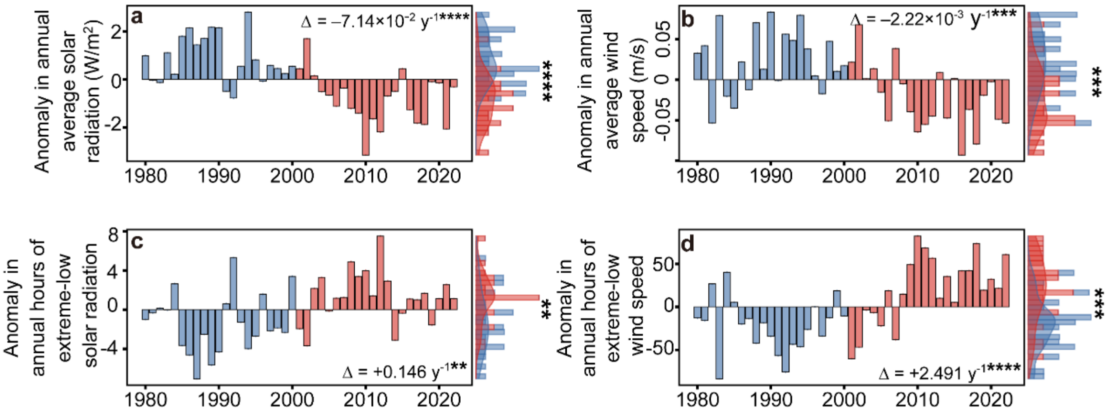

Supplementary Figure 19 | Annual anomalies in climatological variables since 1980. a-b, Anomalies in annual average solar radiation **(a)** and wind speed at hub height **(b)**. **c-d**, Anomalies in annual hours of extremely low solar radiation **(c)** and wind speed at hub height **(d)**. The coefficients indicate robust Theil–Sen's slopes and their corresponding *P* values examined by MK test. Right histograms denote the difference between 1980–2000 and 2001–2022 examined by ANOVA. **, *** and **** represent $P < 0.01$, $P < 0.001$ and $P < 0.0001$, respectively.

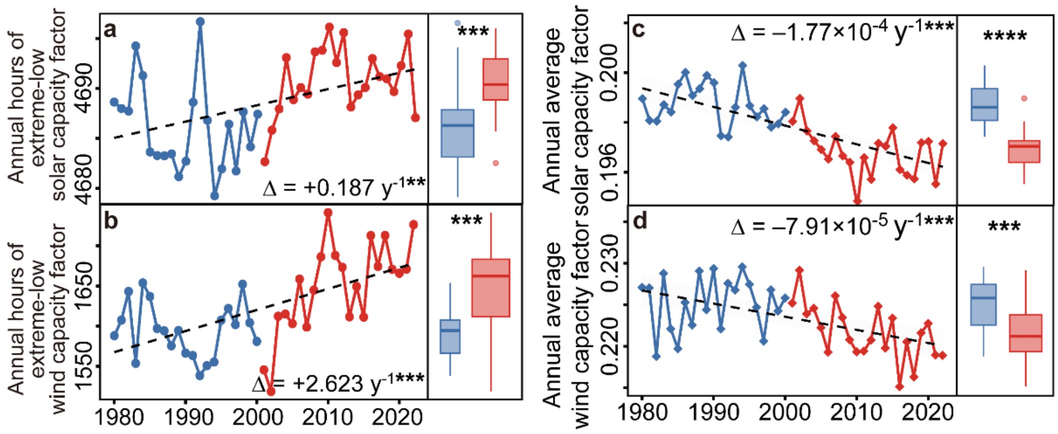

Supplementary Figure 20 | Change in wind and solar capacity factors. Changes in annual hours of extremely low wind **(a)** and solar **(b)** capacity factors. Interannual variability in annual average wind **(c)** and solar **(d)** capacity factors. Extremely low solar and wind capacity factors are defined as below the 10th percentile of daily average value across the 43 surveyed years. The coefficients indicate robust Theil–Sen's slopes and their corresponding *P* values examined by MK test. Right boxplots denote the difference between 1980–2000 and 2001–2022 examined by ANOVA. *** and **** represent $P<0.001$ and $P<0.0001$, respectively.

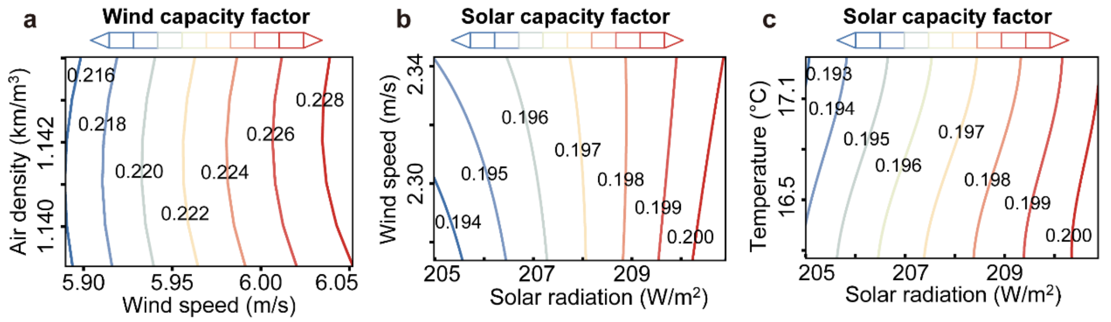

Supplementary Figure 21 | Changes in capacity factors with climatological variables. a, Wind capacity factor is driven largely by wind speed at hub height rather than air density. **b-c**, Solar capacity factor is controlled mostly by surface solar radiation rather than surface wind speed **(b)** and temperature **(c)**.

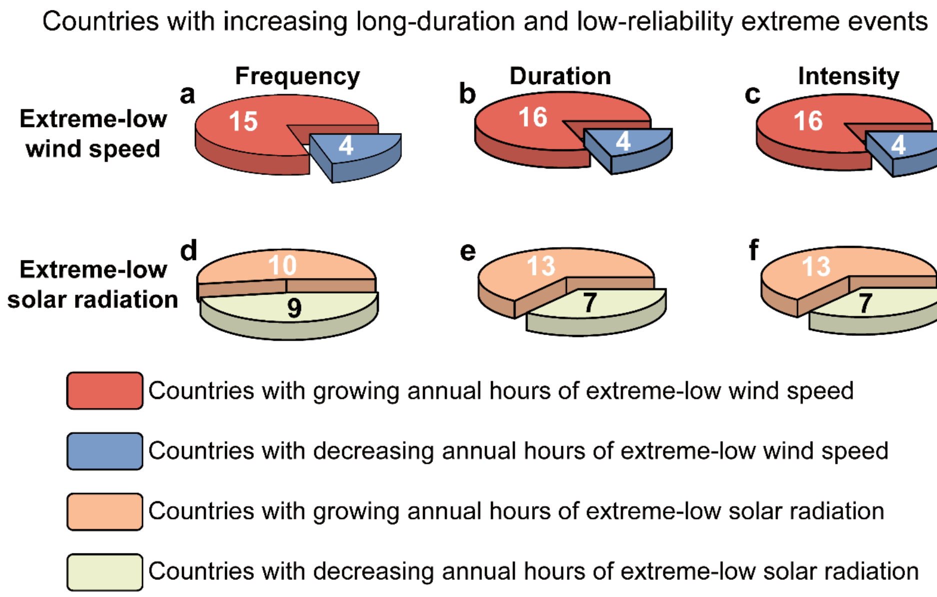

Supplementary Figure 22 | Proportion of countries with increasing extremely low climatological variables to countries with growing extreme power shortage events. a-c, Percentage of countries with growing annual hours of extremely low wind speed to countries with increasing frequency **(a)**, duration **(b)**, and intensity **(c)** of extreme long-duration and low-reliability events. **d-f**, Percentage of countries with growing annual hours of extremely low solar radiation to countries with increasing frequency **(d)**, duration **(e)**, and intensity **(f)** of extreme long-duration and low-reliability events.

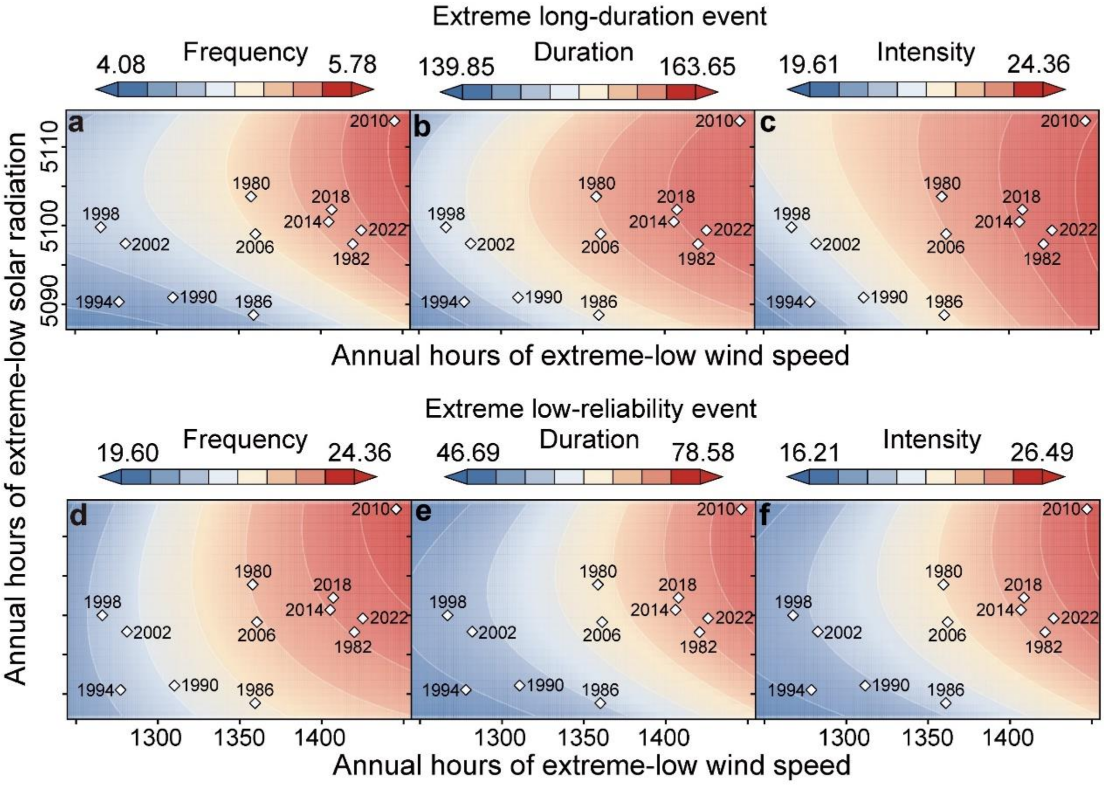

Supplementary Figure 23 | Changes in extreme power shortage events with extremely low wind speed and solar radiation worldwide for the surveyed 178 countries. a-c, Changes in frequency **(a)**, duration **(b)**, and intensity **(c)** of extreme long-duration events with annual hours of extremely low wind speed and solar radiation. **d-f**, Changes in frequency **(d)**, duration **(e)**, and intensity **(f)** of extreme low-reliability events with annual hours of extremely low wind speed and solar radiation.

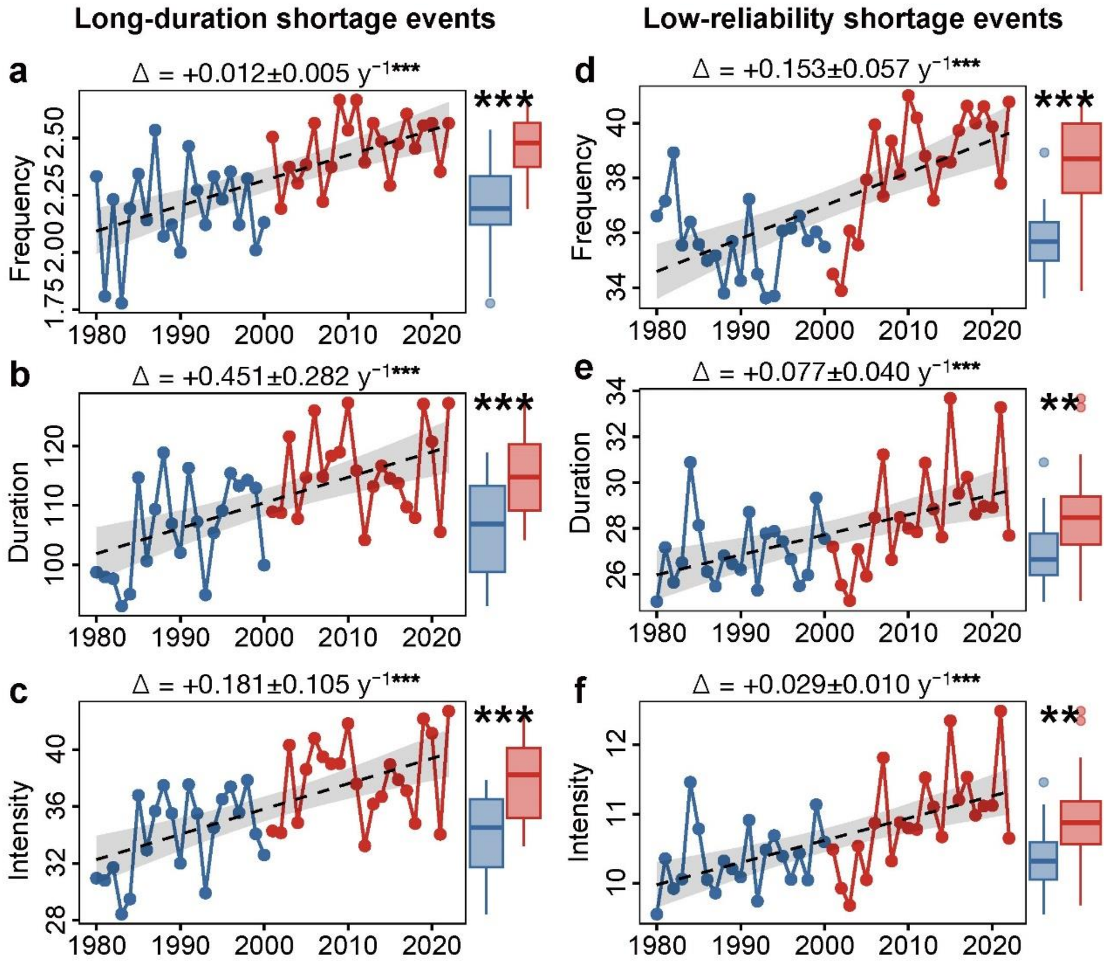

Supplementary Figure 24 | Interannual variability of extreme long-duration events and low-reliability events estimated based upon wind and solar installed capacities predicted by IAMs. a-c, Interannual changes in the frequency **(a)**, duration **(b)**, and intensity **(c)** of extreme long-duration events since 1980. **d-f**, Interannual changes in the frequency **(d)**, duration **(e)**, and intensity **(f)** of extreme low-reliability events since 1980. The coefficients at the top of the panels indicate robust Theil-Sen's slopes and their corresponding *P* values, examined by Mann-Kendall (MK) test. The right boxplots denote the difference before 2000 (blue) and after 2000 (red) examined by analysis of variance (ANOVA). *** and ** represent the significance under the level of $P<0.001$ and $P<0.01$, respectively. Black dashed lines denote linear fitting of annual average values of extreme power shortage events.

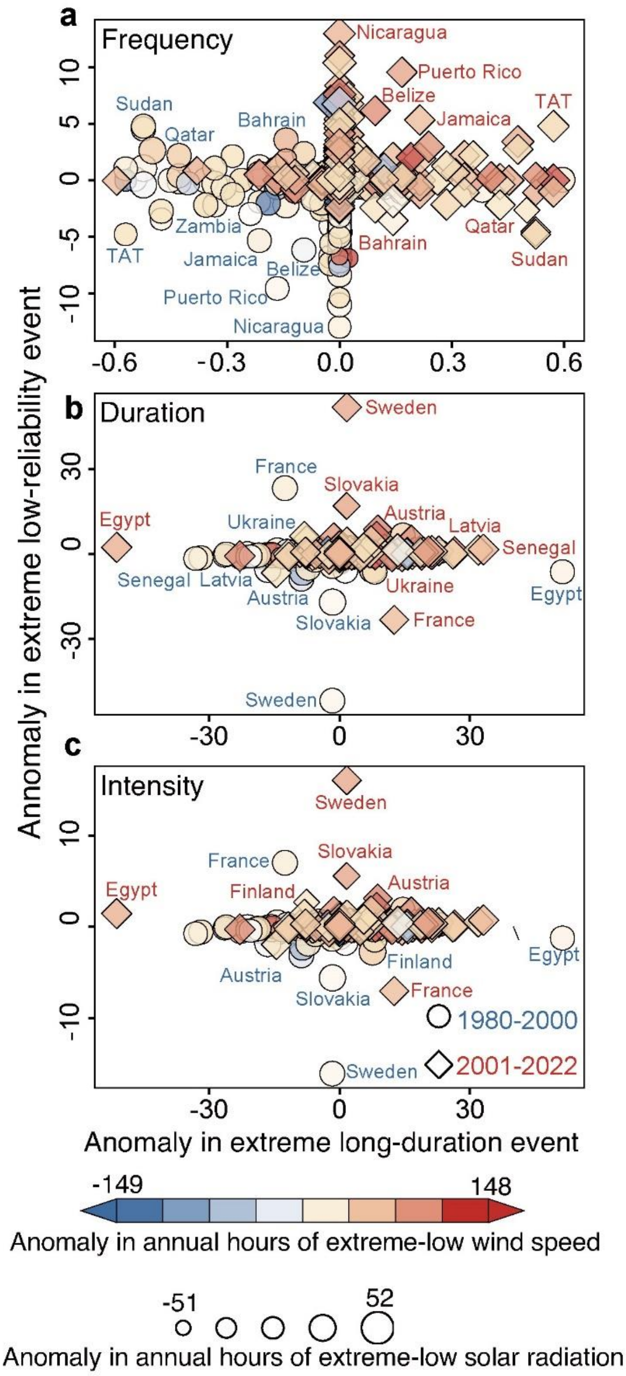

Supplementary Figure 25 | Changes in extreme power shortage events with extremely low wind speed and solar radiation based on wind and solar installed capacities predicted by IAMs. a-c, Relationship between anomalies in frequency **(a)**, duration **(b)**, and intensity **(c)** of extreme power shortage events and anomalies in annual hours of extremely low solar radiation and wind speed at hub height for individual countries.

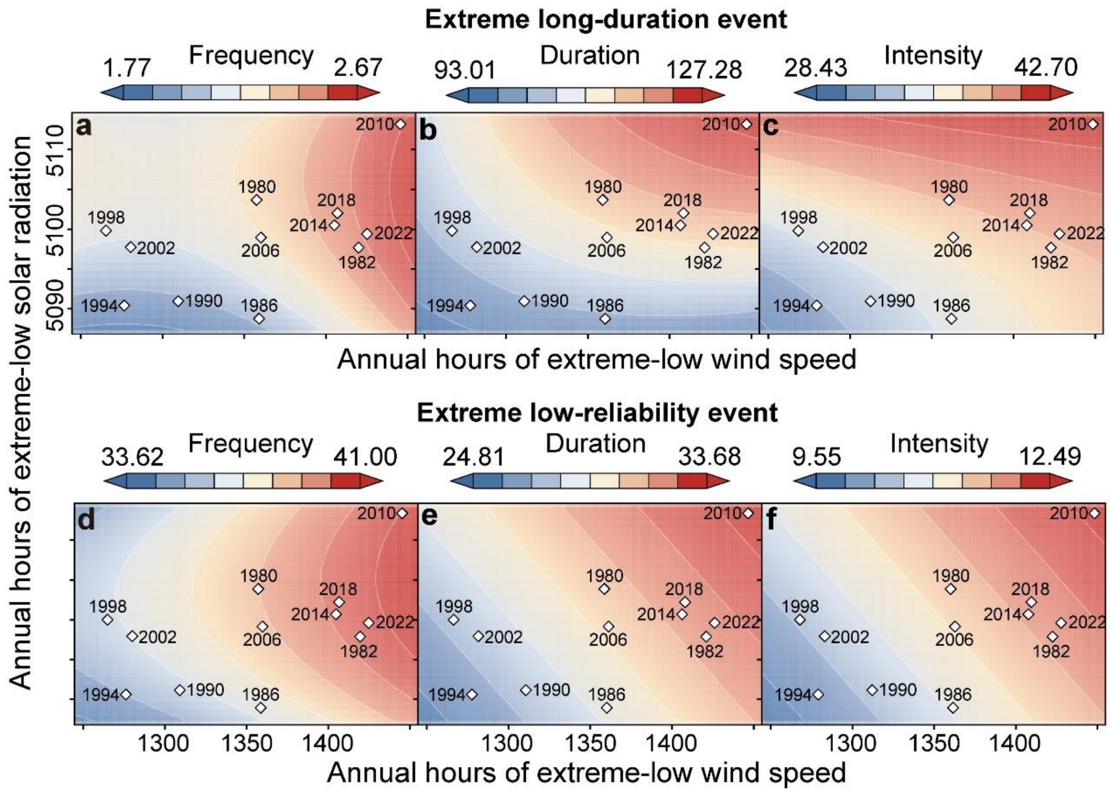

Supplementary Figure 26 | Changes in extreme power shortage events with extremely low wind speed and solar radiation worldwide based upon wind and solar installed capacities predicted by IAMs. a-c, Changes in frequency **(a)**, duration **(b)**, and intensity **(c)** of extreme long-duration events with annual hours of extremely low wind speed and solar radiation. **d-f**, Changes in frequency **(d)**, duration **(e)**, and intensity **(f)** of extreme low-reliability events with annual hours of extremely low wind speed and solar radiation.

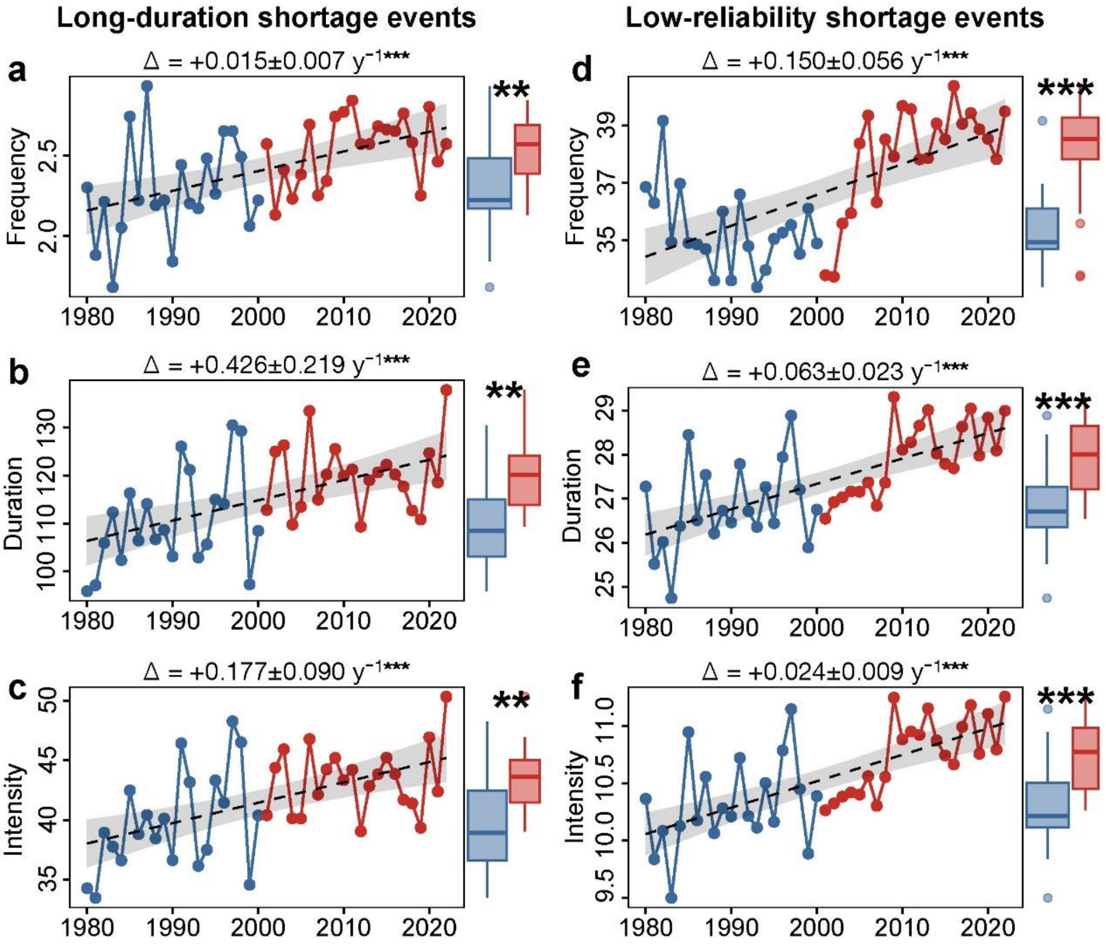

Supplementary Figure 27 | Interannual variability of extreme long-duration events and low-reliability events estimated based upon wind/solar ratio predicted by IAMs. a-c, Interannual changes in the frequency **(a)**, duration **(b)**, and intensity **(c)** of extreme long-duration events since 1980. **d-f**, Interannual changes in the frequency **(d)**, duration **(e)**, and intensity **(f)** of extreme low-reliability events since 1980. The coefficients at the top of the panels indicate robust Theil-Sen's slopes and their corresponding *P* values, examined by Mann-Kendall (MK) test. The right boxplots denote the difference before 2000 (blue) and after 2000 (red) examined by analysis of variance (ANOVA). *** and ** represent the significance under the level of P<0.001 and P<0.01, respectively. Black dashed lines denote linear fitting of annual average values of extreme power shortage events.

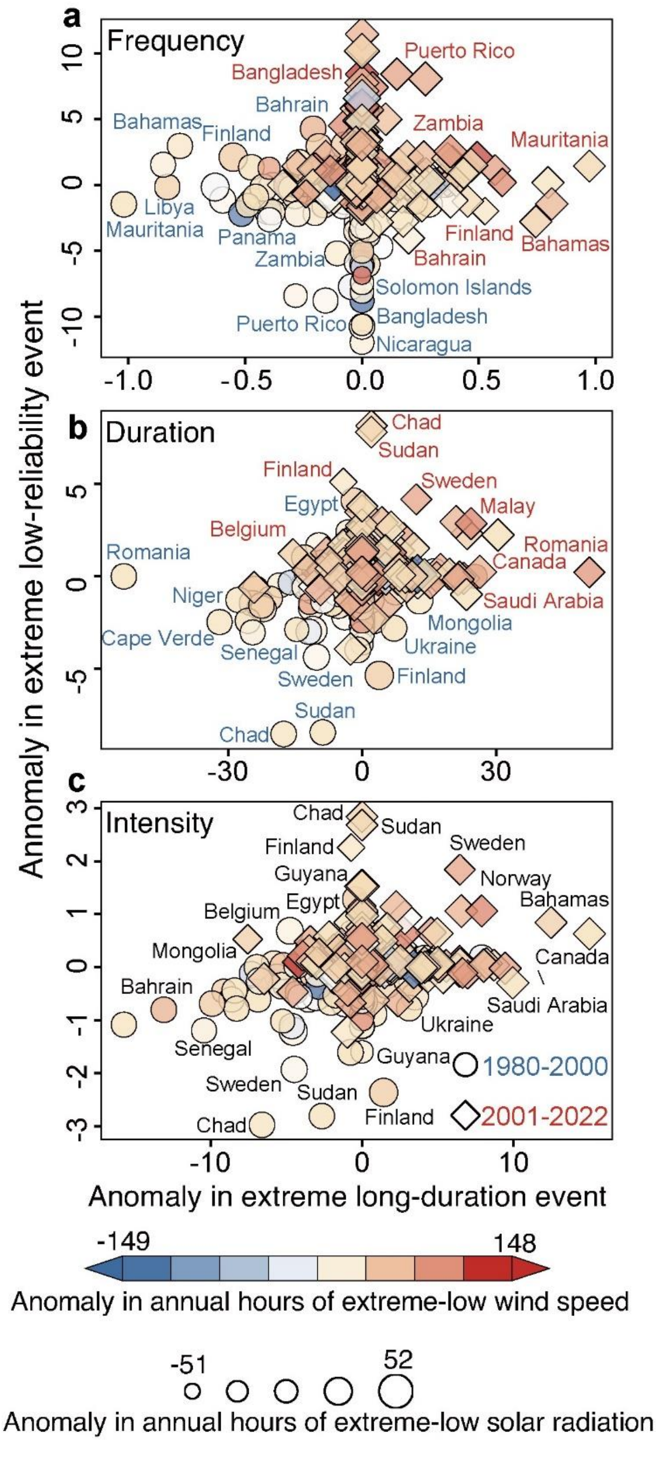

Supplementary Figure 28 | Changes in extreme power shortage events with extremely low wind speed and solar radiation based upon wind/solar ratio predicted by IAMs. a-c, Relationship between anomalies in frequency **(a)**, duration **(b)**, and intensity **(c)** of extreme power shortage events and anomalies in annual hours of extremely low solar radiation and wind speed at hub height for individual countries.

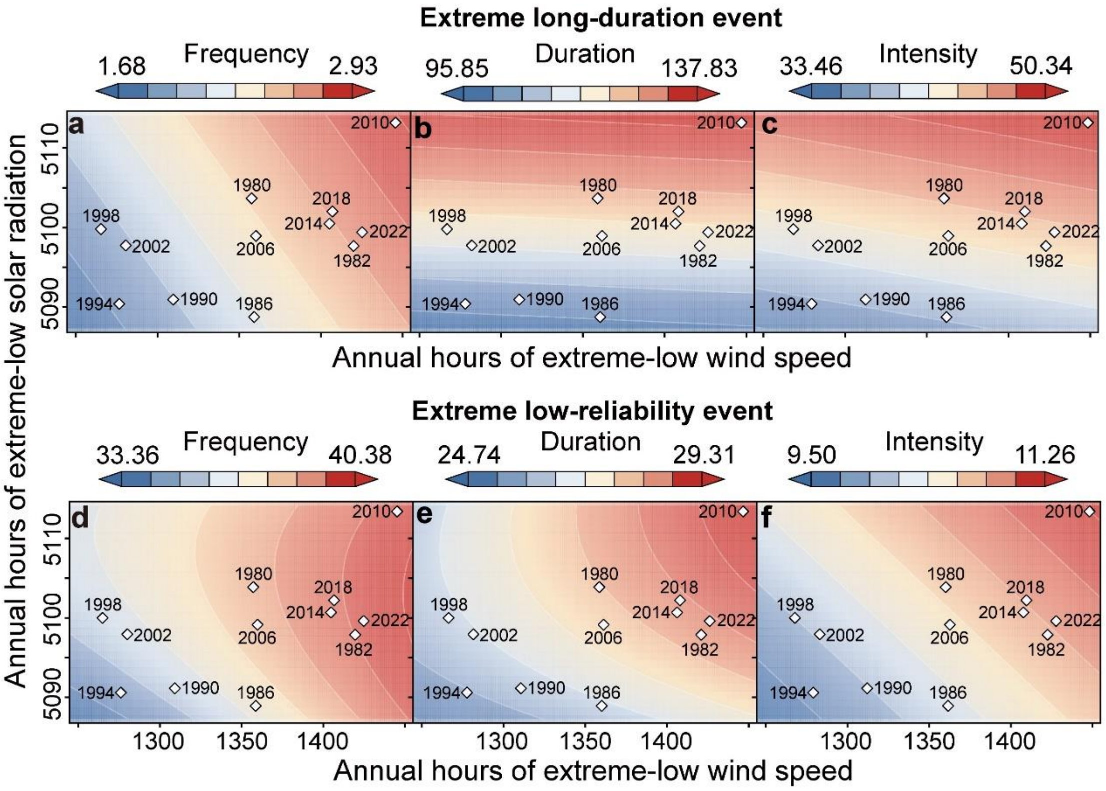

Supplementary Figure 29 | Changes in extreme power shortage events with extremely low wind speed and solar radiation worldwide based upon wind/solar ratio predicted by IAMs. a-c, Changes in frequency **(a)**, duration **(b)**, and intensity **(c)** of extreme long-duration events with annual hours of extremely low wind speed and solar radiation. **d-f**, Changes in frequency **(d)**, duration **(e)**, and intensity **(f)** of extreme low-reliability events with annual hours of extremely low wind speed and solar radiation.

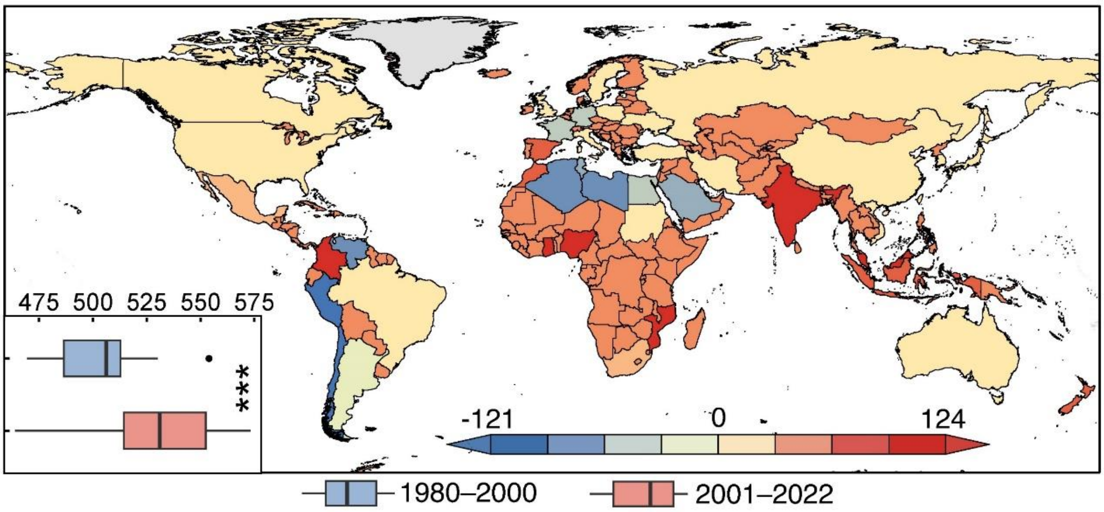

Supplementary Figure 30 | Changes in annual hours of compound extremely low wind speed and solar radiation events for individual countries between 1980–2000 and 2001–2022. The left-bottom boxplots denote the difference of compound extremely low wind speed and solar radiation events for individual countries between 1980–2000 (blue) and 2000–2022 (red) examined by one-way analysis of variance (ANOVA), where *** represent the significance under the level of $P<0.001$.

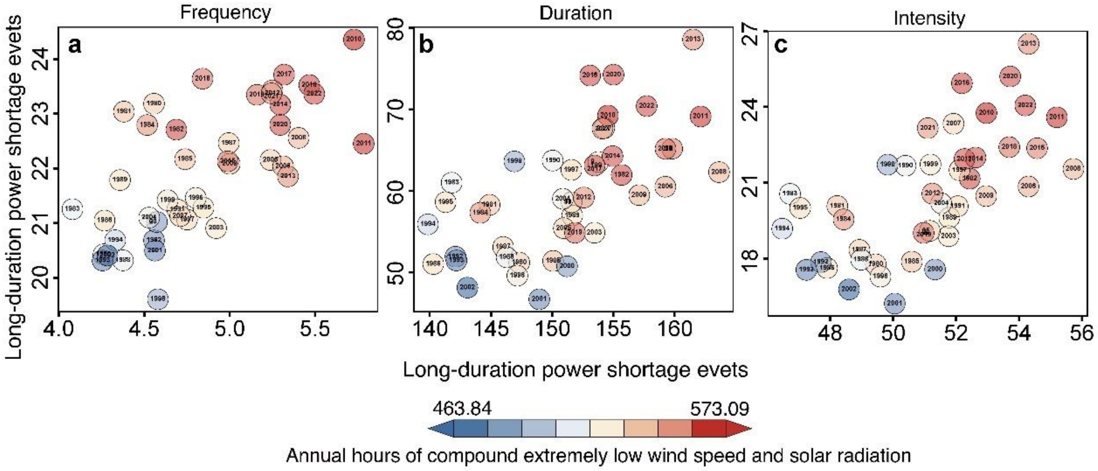

Supplementary Figure 31 | Relationship between extreme power shortage events and compound extremely low wind speed and solar radiation. a-c, Relationship between annual average frequency **(a)**, duration **(b)**, and intensity **(c)** of extreme power shortage events and annual average hours of compound extremely low wind speed and solar radiation between 1980 and 2022.

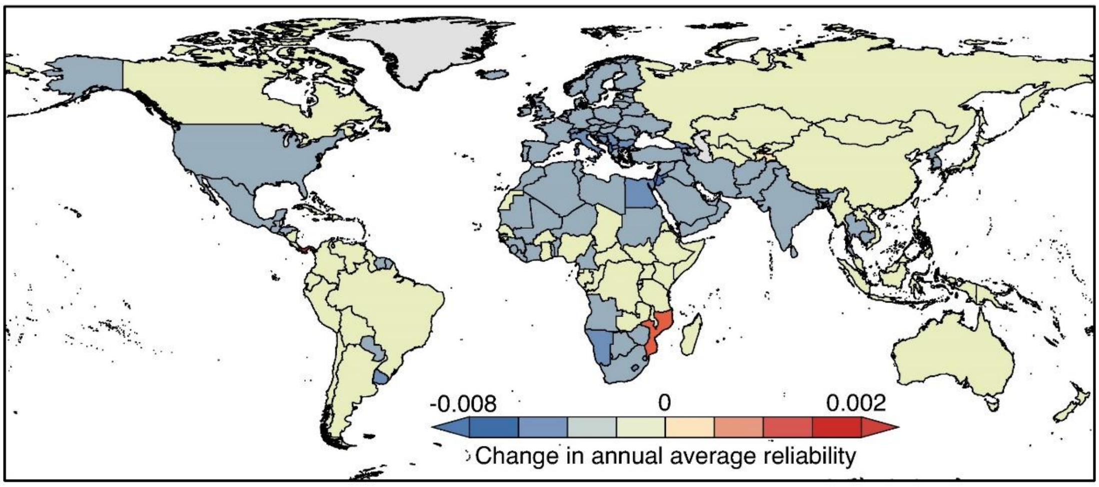

Supplementary Figure 32 | The impacts of solar tracking systems on system reliability across the world. The changes in annual average reliability between single-axis and dura-axis solar tracking systems during the period of 1980 and 2000.

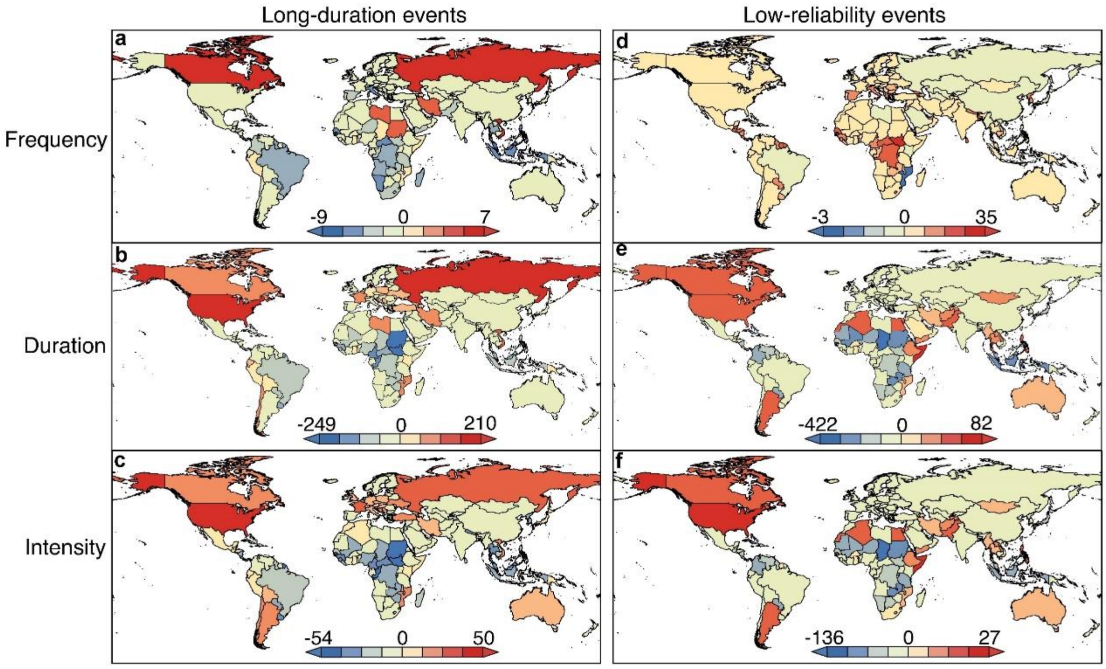

Supplementary Figure 33 | The impacts of solar tracking systems on extreme power shortage events across the world. a-c, The changes in frequency **(a)**, duration **(b)**, intensity **(c)** of long-duration events between single-axis and dura-axis solar tracking systems during the period of 1980 and 2000. **d-f**, The changes in frequency **(d)**, duration **(e)**, intensity **(f)** of low-reliability events between single-axis and dura-axis solar tracking systems during the period of 1980 and 2000.

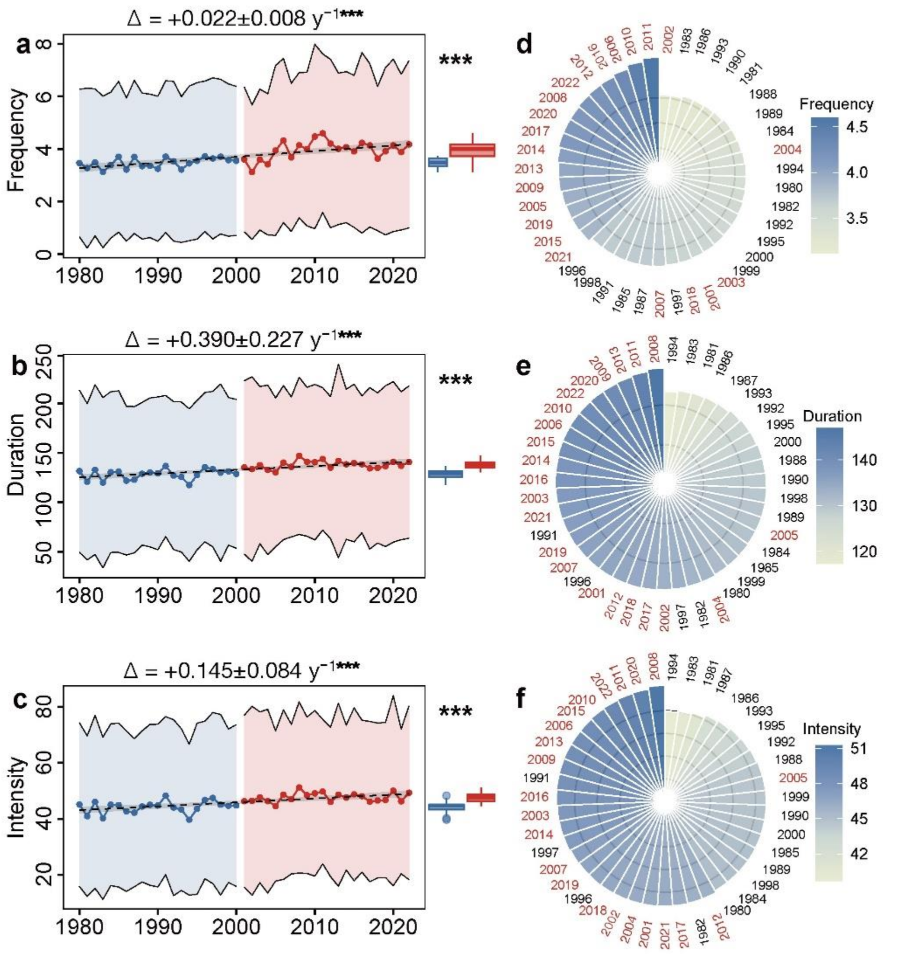

Supplementary Figure 34 | Interannual variability of extreme long-duration events with consideration of solar tracking system progress. a-c, Interannual changes in the frequency **(a)**, duration **(b)**, and intensity **(c)** of extreme long-duration events since 1980. The coefficients at the top of the panels indicate robust Theil-Sen's slopes and their corresponding *P* values, examined by Mann-Kendall (MK) test. The right boxplots denote the difference before 2000 (blue) and after 2000 (red) examined by analysis of variance (ANOVA). *** represents the significance under the level of $P<0.001$. Black dashed lines denote linear fitting of annual average values across 178 countries. **d-f**, Rank ordering of the annual average frequency **(d)**, duration **(e)**, and intensity **(f)** of extreme long-duration events across the surveyed 43 years, in which years between 1980 and 2000 are labeled with black and those between 2001 and 2022 are labeled with red.

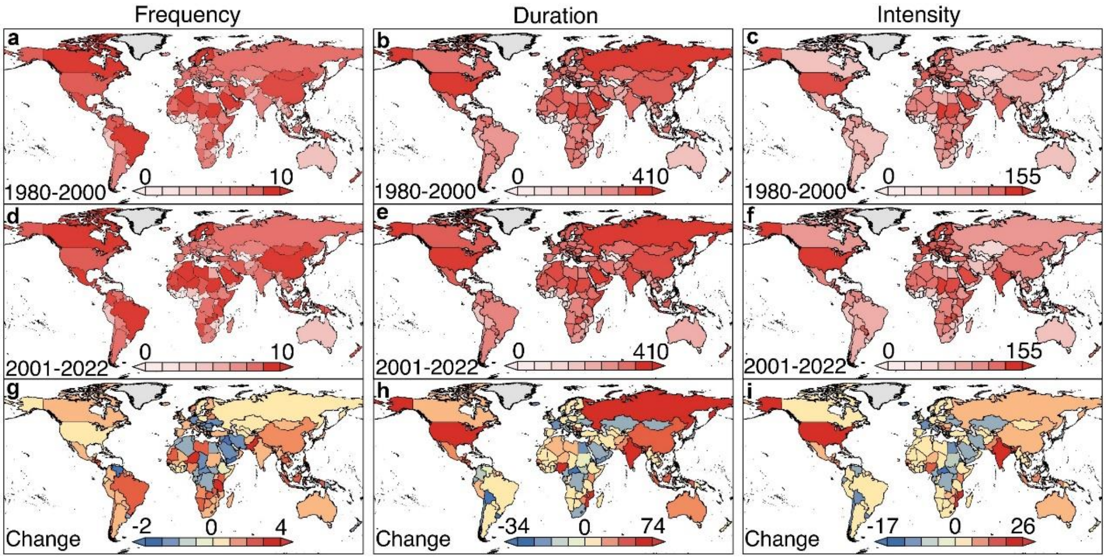

Supplementary Figure 35 | Maps of extreme long-duration events with consideration of solar tracking system progress. a-c, Annual average frequency **(a)**, duration **(b)**, and intensity **(c)** of extreme long-duration events during the period 1980–2000. **d-f**, Annual average frequency **(d)**, duration **(e)**, and intensity **(f)** of extreme long-duration events during the period 2001–2022. **g-i**, Changes in annual average frequency **(g)**, duration **(h)**, and intensity **(i)** of extreme long-duration events between 1980–2000 and 2001–2022.

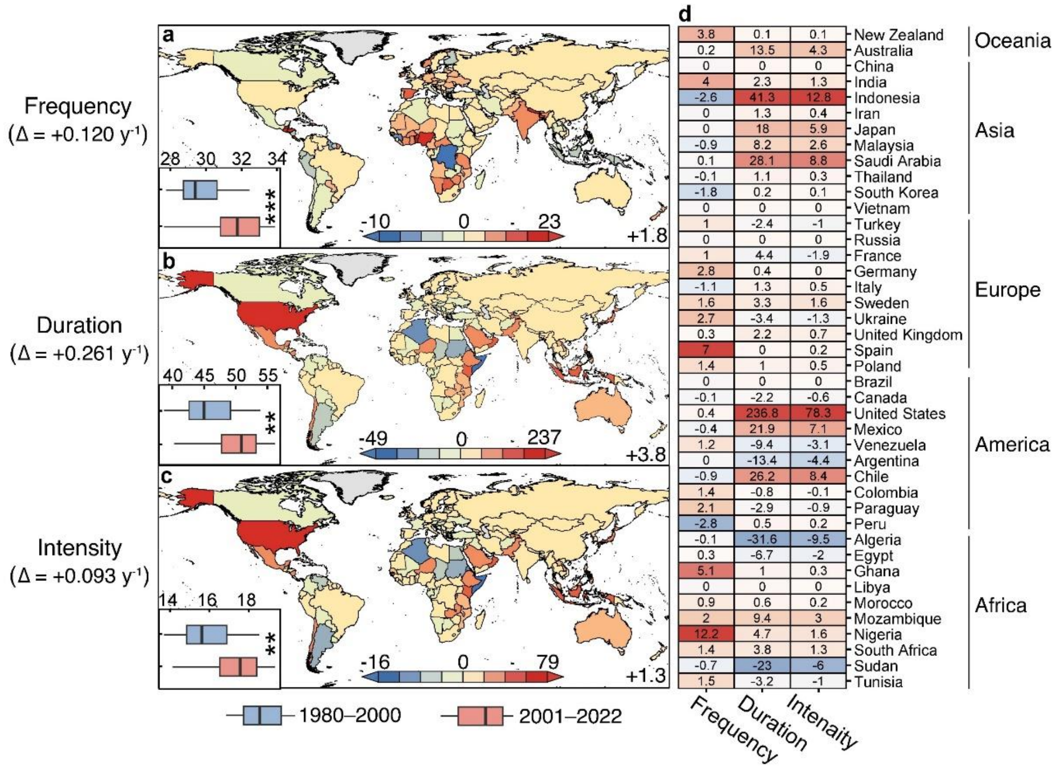

Supplementary Figure 36 | Changes in extreme low-reliability events with consideration of solar tracking system progress. a-c, Changes in annual average frequency **(a)**, duration **(b)**, and intensity **(c)** of extreme low-reliability events between 1980–2000 and 2001–2022. The left-bottom boxplots denote the difference between 1980–2000 (blue) and 2000–2022 (red) examined by ANOVA, where ** and *** represent the significance under the level of P<0.01 and P<0.001, respectively. **d**, Changes in extreme low-reliability events for 42 major countries between 1980–2000 and 2001–2022.

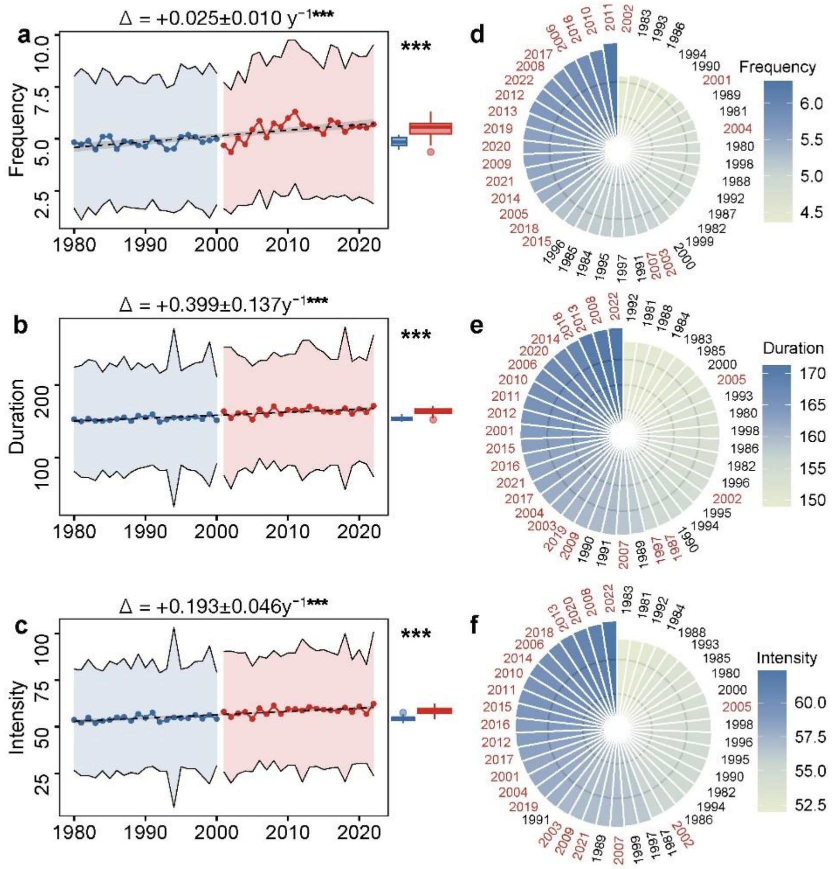

Supplementary Figure 37 | Interannual variability of extreme long-duration events of wind-solar supply system with consideration of CSP technology. a-c, Interannual changes in the frequency **(a)**, duration **(b)**, and intensity **(c)** of extreme long-duration events since 1980. The coefficients at the top of the panels indicate robust Theil-Sen's slopes and their corresponding *P* values, examined by Mann-Kendall (MK) test. The right boxplots denote the difference before 2000 (blue) and after 2000 (red) examined by analysis of variance (ANOVA). *** represents the significance under the level of $P<0.001$. Black dashed lines denote linear fitting of annual average values across 178 countries. **d-f**, Rank ordering of the annual average frequency **(d)**, duration **(e)**, and intensity **(f)** of extreme long-duration events across the surveyed 43 years, in which years between 1980 and 2000 are labeled with black and those between 2001 and 2022 are labeled with red.

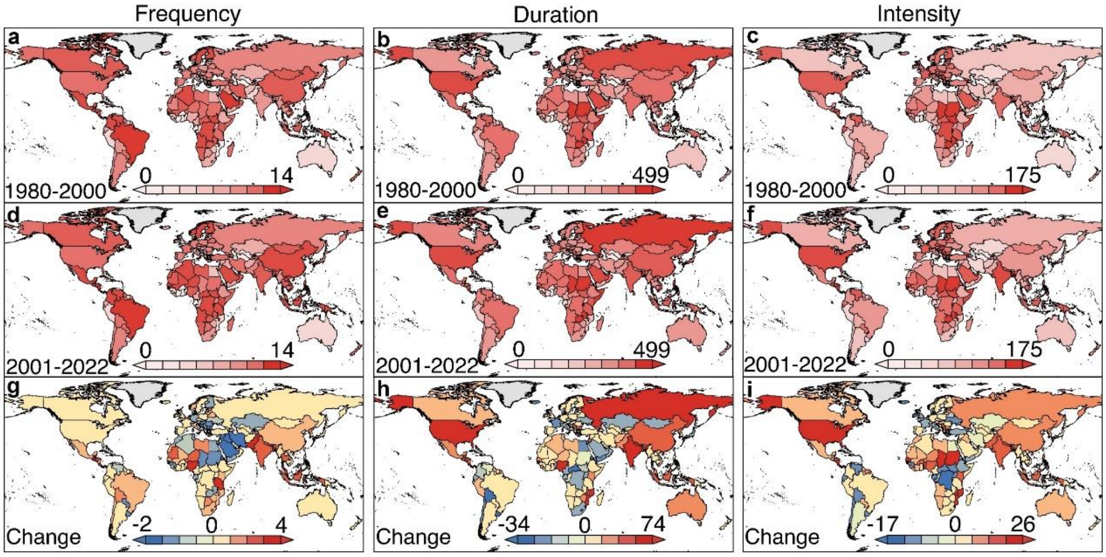

Supplementary Figure 38 | Maps of extreme long-duration events with CSP technology during the period 1980–2000 and 2001–2022. a-c, Annual average frequency **(a)**, duration **(b)**, and intensity **(c)** of extreme long-duration events during the period 1980–2000. **d-f**, Annual average frequency **(d)**, duration **(e)**, and intensity **(f)** of extreme long-duration events during the period 2001–2022. **g-i**, Changes in annual average frequency **(g)**, duration **(h)**, and intensity **(i)** of extreme long-duration events between 1980–2000 and 2001–2022.

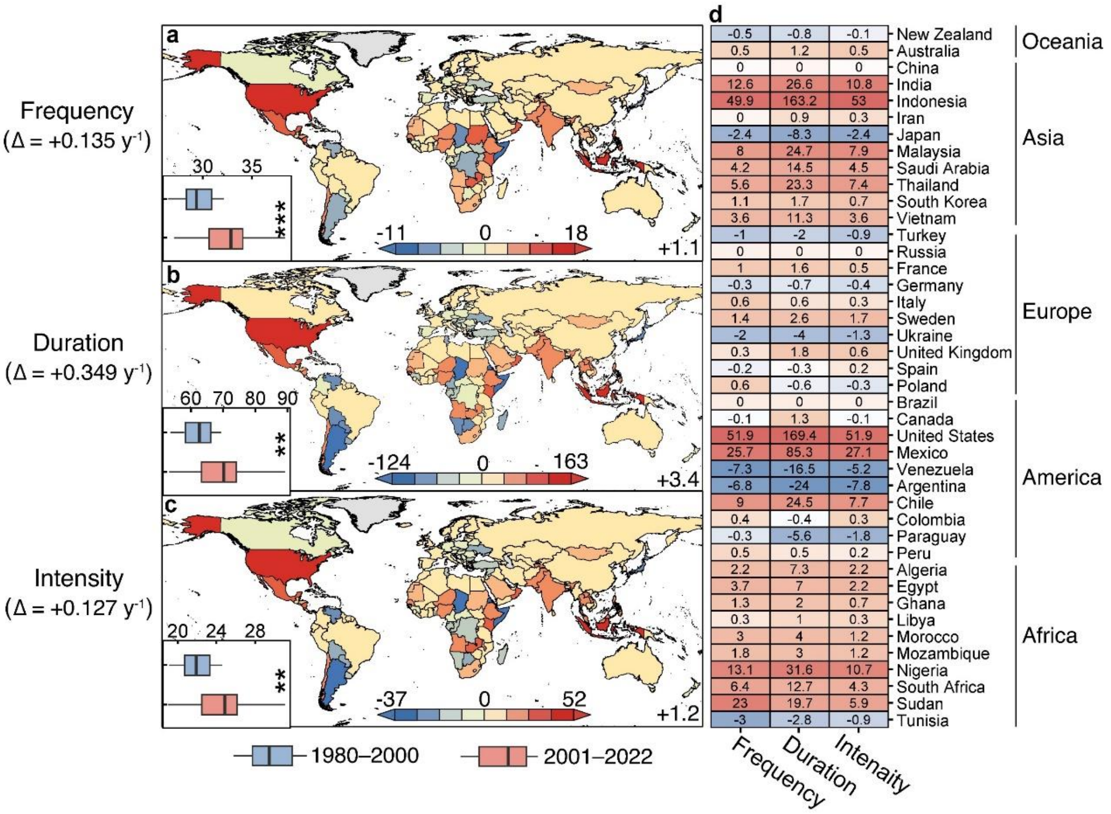

Supplementary Figure 39 | Changes in extreme low-reliability events with CSP technology since 1980. a-c, Changes in annual average frequency **(a)**, duration **(b)**, and intensity **(c)** of extreme low-reliability events between 1980–2000 and 2001–2022. The left-bottom boxplots denote the difference between 1980–2000 (blue) and 2000–2022 (red) examined by ANOVA, where ** and *** represent the significance under the level of P<0.01 and P<0.001, respectively. **d**, Changes in extreme low-reliability events for 42 major countries between 1980–2000 and 2001–2022.

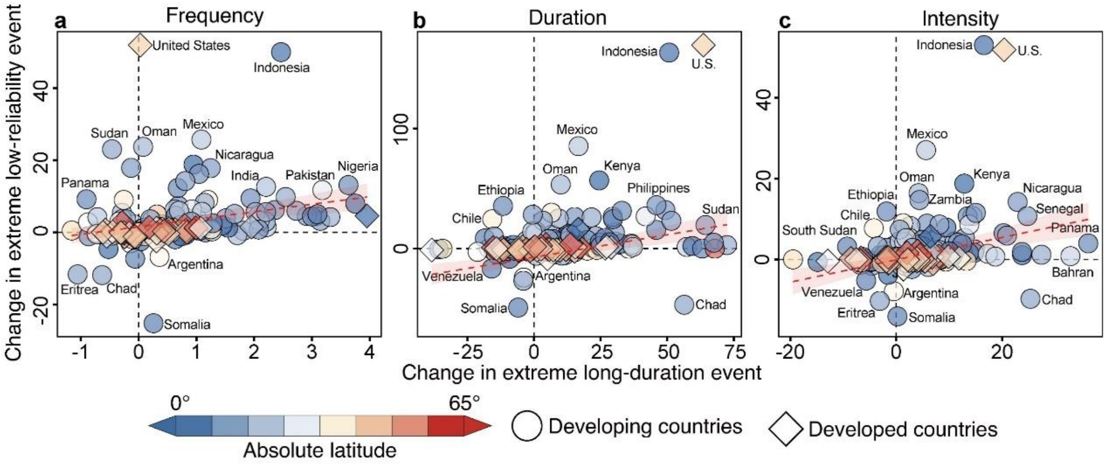

Supplementary Figure 40 | Relationship of changes in extreme long-duration and low-reliability events with consideration of CSP technology. Changes in frequency **(a)**, duration **(b)**, and intensity **(c)** of extreme long-duration and low-reliability events for the surveyed 178 countries between 1980–2000 and 2001–2022. Dashed red lines with shallow red shading are linear fittings between the two types of events and their 95% confidence intervals.

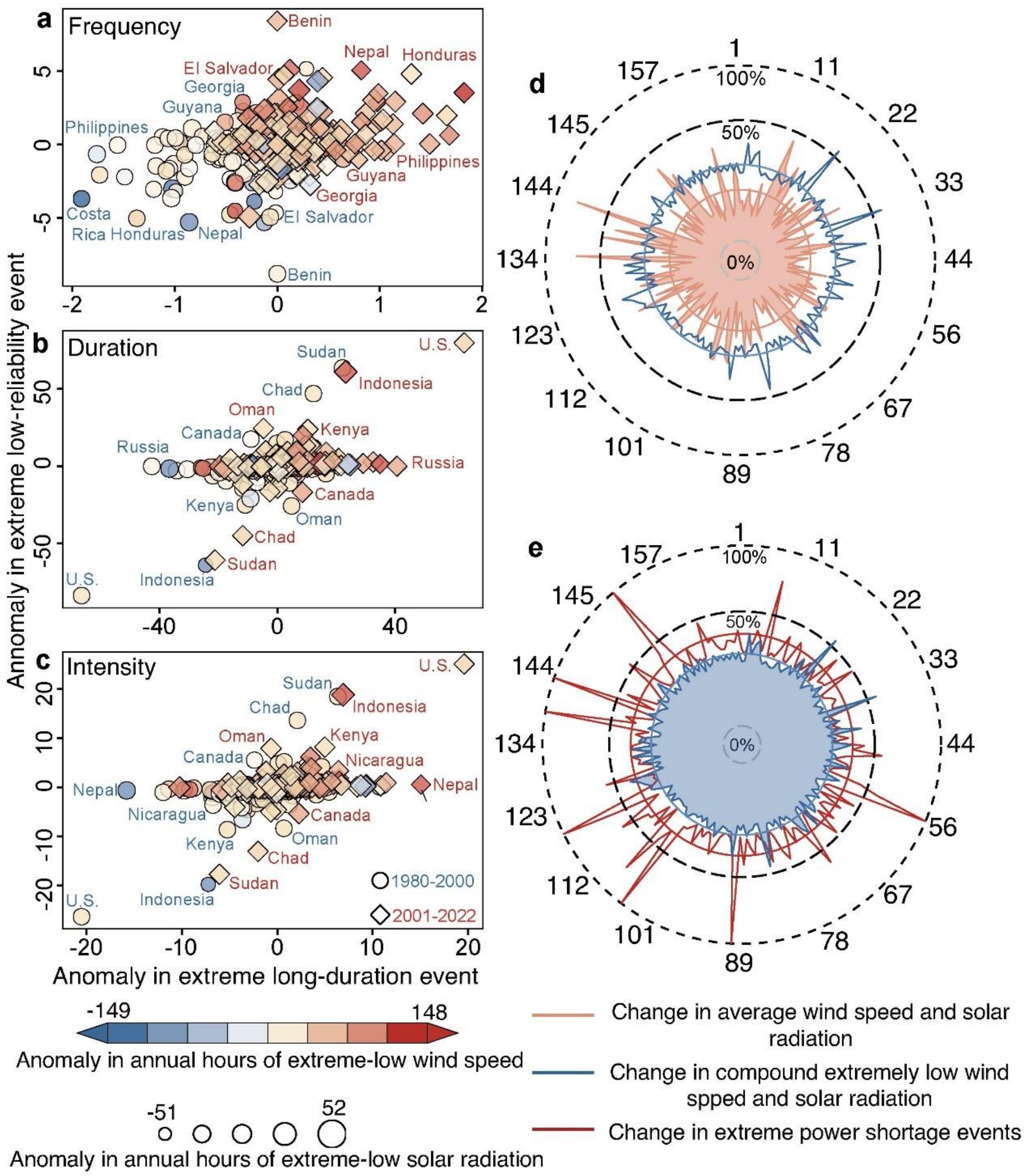

Supplementary Figure 41 | Changes in extreme power shortage events with extremely low wind speed and solar radiation with consideration of CSP technology. a-c, Relationship between anomalies in frequency **(a)**, duration **(b)**, and intensity **(c)** of extreme power shortage events and anomalies in annual hours of extremely low solar radiation and wind speed at hub height for the 178 surveyed countries. **d**, Relationship between change in annual average wind speed and change in annual hours of compound extremely low wind speed & solar radiation between 1980–2000 and 2001–2022. **e**, Relationship between the relative change in annual hours of extremely low wind speed & solar radiation and the relative change in extreme power shortage events between 1980–2000 and 2001–2022. The relative change above 100% is visualized as 100% change. The lines and circles denote individual country's change and the average changes across 178 surveyed countries, respectively. The surrounding digits represent the order number of the 178 countries that are listed in Supplementary Table 7.

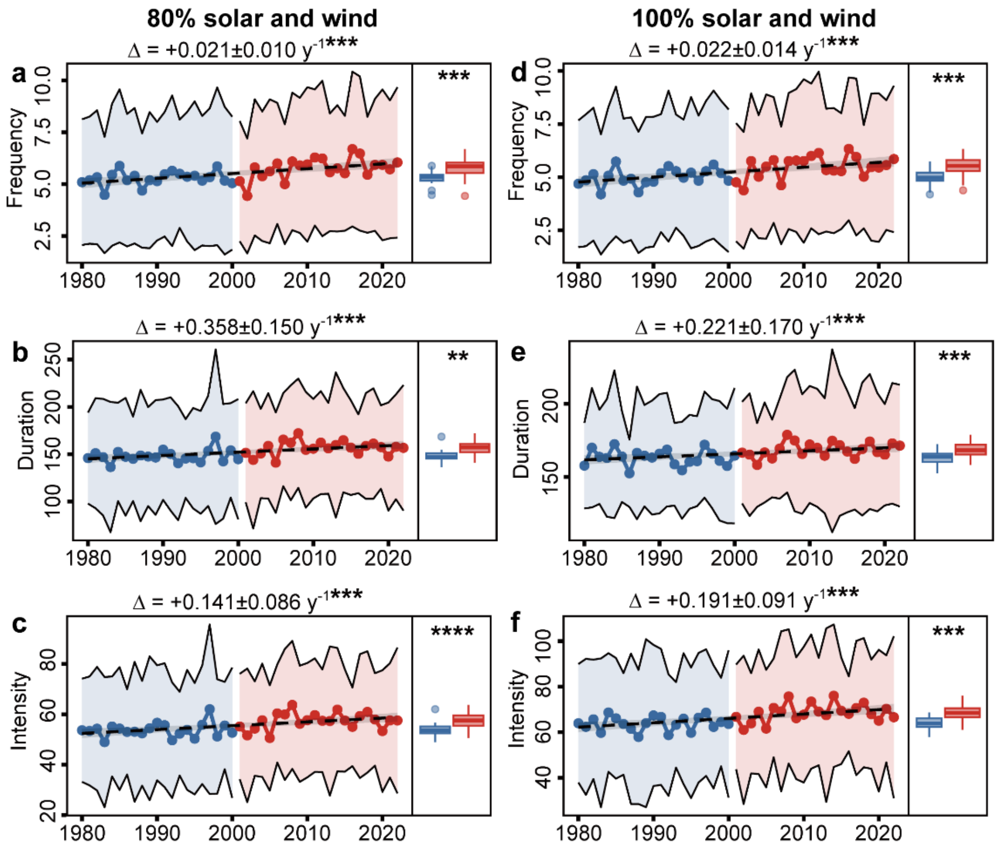

Supplementary Figure 42 | Sensitivity tests for changes in extreme long-duration events under 80% and 100% wind-solar electricity systems. a-c, Changes in frequency **(a)**, duration **(b)**, and intensity **(c)** of extreme long-duration events in the system where 80% electricity demand is satisfied by wind and solar energies. **d-f**, Changes in frequency **(d)**, duration **(e)**, and intensity **(f)** of extreme long-duration events where 100% electricity demand is satisfied by wind and solar energies. The coefficients at the top of the panels indicate robust Theil-Sen's slopes and their corresponding *P* values, examined by MK test. The right boxplots denote the difference before 2000 (blue) and after 2000 (red) examined by ANOVA. *, **, and *** represent the significance under the level of P<0.05, P<0.01, and P<0.001, respectively. Black dashed lines with shading denote linear fitting and the standard deviation of annual average values across 42 countries.

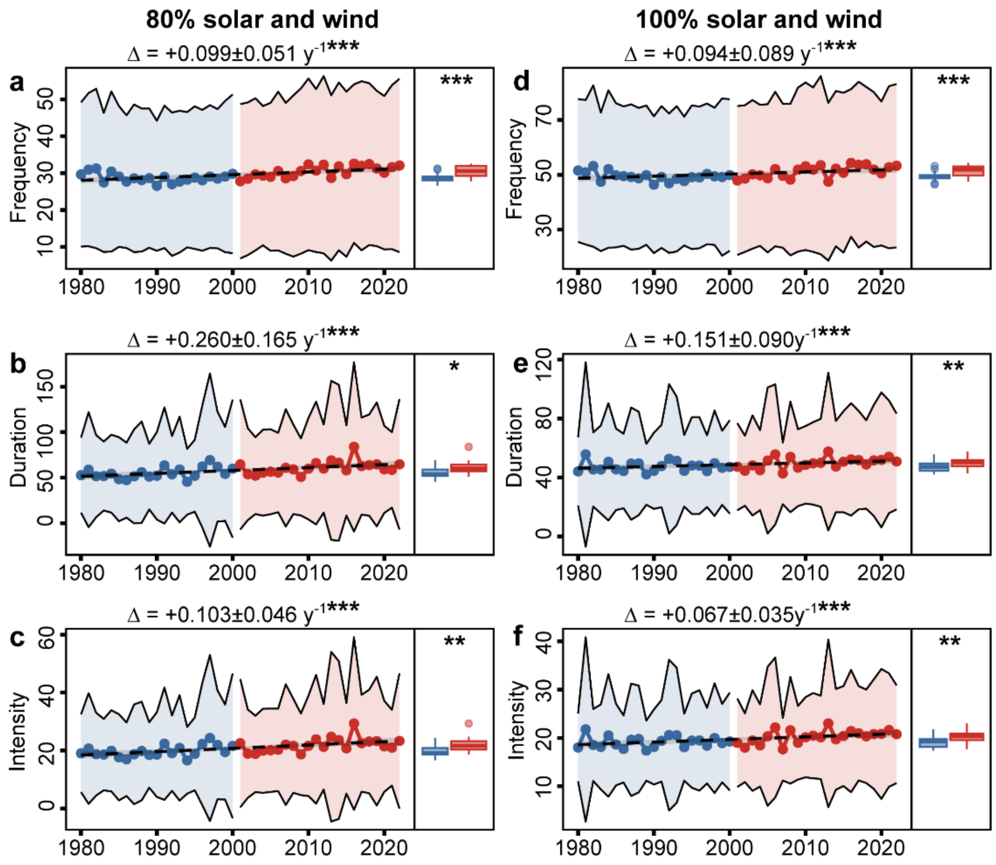

Supplementary Figure 43 | Sensitivity tests for changes in extreme low-reliability events under 80% and 100% wind-solar electricity systems. a-c, Changes in frequency **(a)**, duration **(b)**, and intensity **(c)** of extreme low-reliability events in the system where 80% electricity demand is satisfied by wind and solar energies. **d-f**, Changes in frequency **(d)**, duration **(e)**, and intensity **(f)** of extreme low-reliability events where 100% electricity demand is satisfied by wind and solar energies. In 80% and 100% wind-solar system, we make more strict definition of extreme low-reliability events when demand is unmet for at least 18 hours with over 30% power gap. The coefficients at the top of the panels indicate robust Theil–Sen's slopes and their corresponding *P* values, examined by MK test. The right boxplots denote the difference between 1980–2000 (blue) and 2000–2022 (red) examined by ANOVA. *, **, and *** represent the significance under the level of $P<0.05$, $P<0.01$, and $P<0.001$, respectively. Black dashed lines denote linear fitting of annual average values across 42 countries.

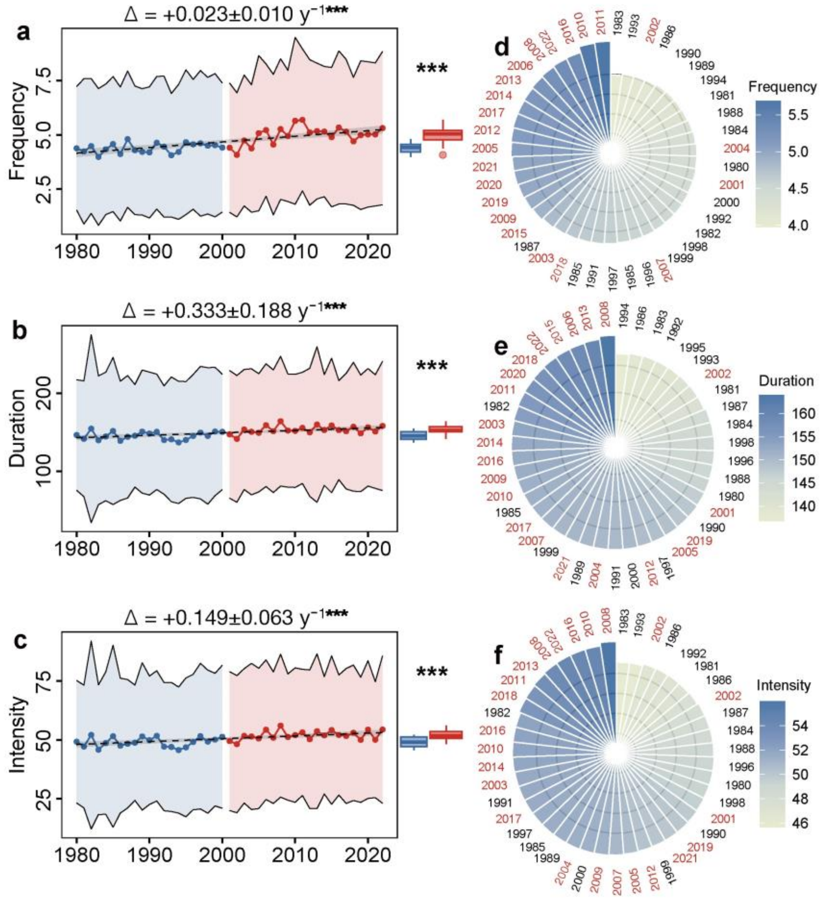

Supplementary Figure 44 | Interannual variability in extreme long-duration events of wind-solar supply system using the wind-solar shares predicted by REMIND model. a-c, Interannual changes in the frequency **(a)**, duration **(b)**, and intensity **(c)** of extreme long-duration events since 1980. The coefficients at the top of the panels indicate robust Theil-Sen's slopes and their corresponding P values, examined by Mann-Kendall (MK) test. The right boxplots denote the difference before 2000 (blue) and after 2000 (red) examined by analysis of variance (ANOVA). *** represents the significance under the level of P<0.001. Black dashed lines denote linear fitting of annual average values across 178 countries. **d-f**, Rank ordering of the annual average frequency **(d)**, duration **(e)**, and intensity **(f)** of extreme long-duration events across the surveyed 43 years, in which years between 1980 and 2000 are labeled with black and those between 2001 and 2022 are labeled with red.

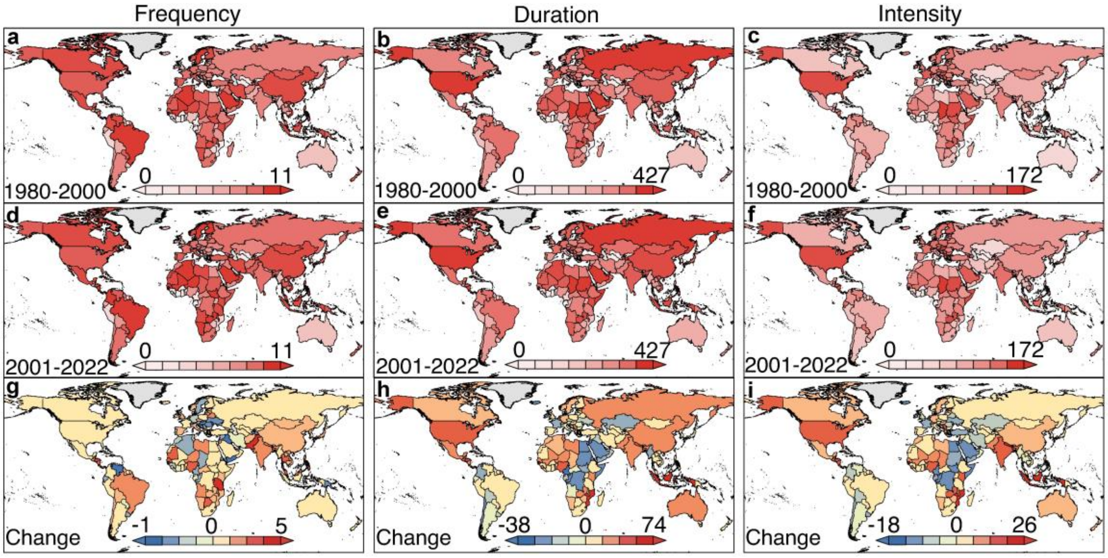

Supplementary Figure 45 | Maps of extreme long-duration events based upon the wind-solar shares predicted by REMIND model. a-c, Annual average frequency **(a)**, duration **(b)**, and intensity **(c)** of extreme long-duration events during the period 1980–2000. **d-f**, Annual average frequency **(d)**, duration **(e)**, and intensity **(f)** of extreme long-duration events during the period 2001–2022. **g-i**, Changes in annual average frequency **(g)**, duration **(h)**, and intensity **(i)** of extreme long-duration events between 1980–2000 and 2001–2022.

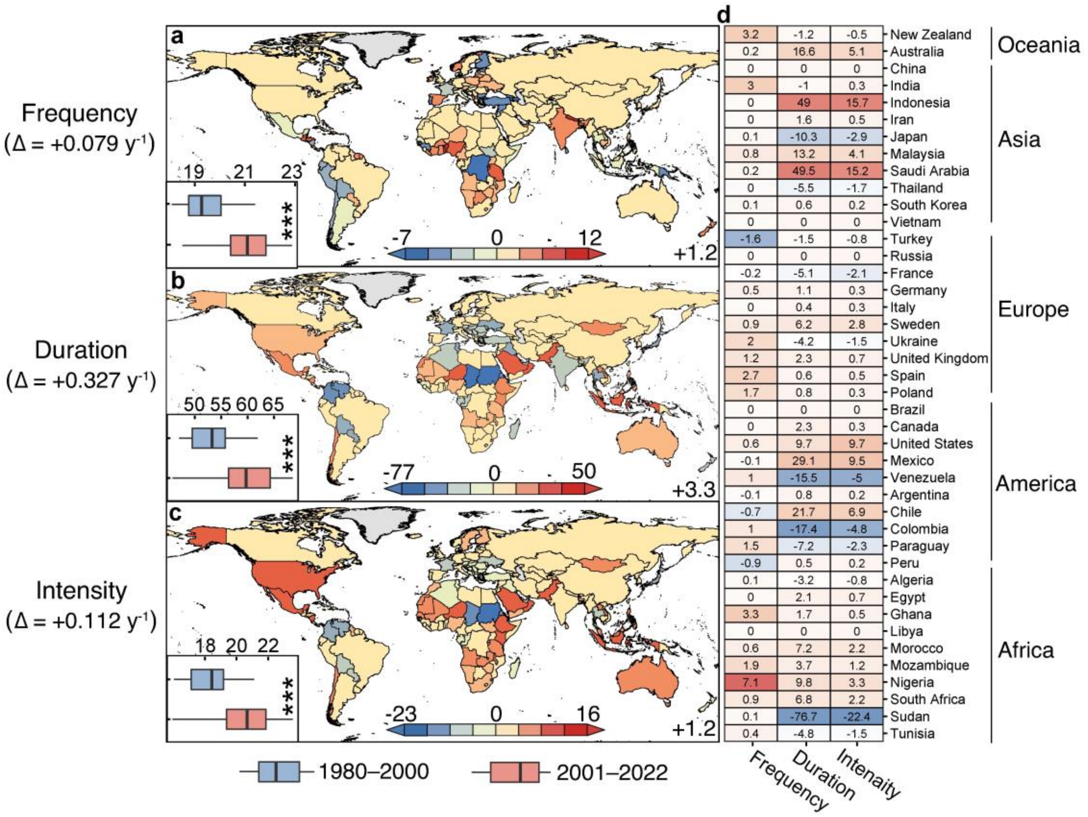

Supplementary Figure 46 | Changes in extreme low-reliability events calculated through the wind-solar shares predicted by REMIND model. a-c, Changes in annual average frequency **(a)**, duration **(b)**, and intensity **(c)** of extreme low-reliability events between 1980–2000 and 2001–2022. The left-bottom boxplots denote the difference between 1980–2000 (blue) and 2000–2022 (red) examined by ANOVA, where *** represent the significance under the level of P<0.001. **d**, Changes in extreme low-reliability events for 42 major countries between 1980–2000 and 2001–2022.

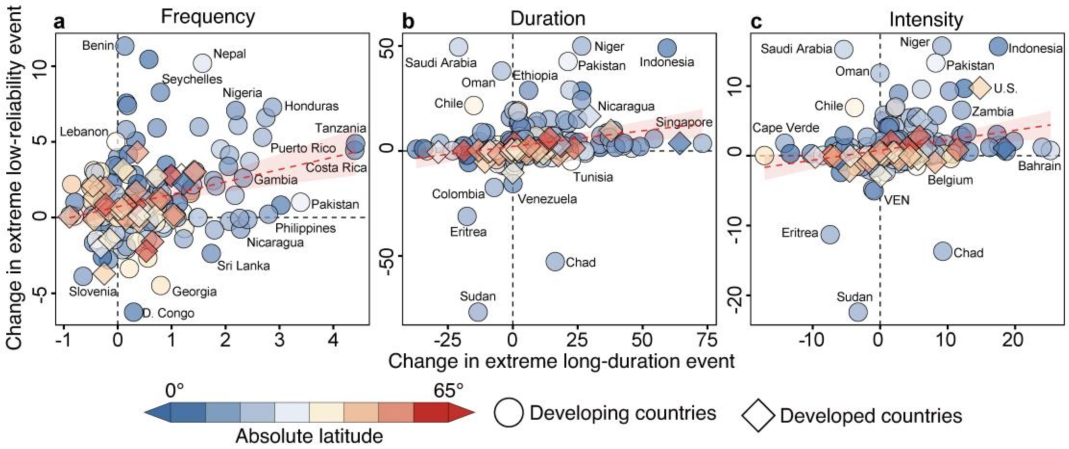

Supplementary Figure 47 | Relationship of changes in extreme long-duration and low-reliability events using the wind-solar shares predicted by REMIND model. Changes in frequency **(a)**, duration **(b)**, and intensity **(c)** of extreme long-duration and low-reliability events for the surveyed 178 countries between 1980–2000 and 2001–2022. Dashed red lines with shallow red shading are linear fittings between the two types of and their 95% confidence intervals. VEN and D. Congo denote Venezuela and Democratic Republic of the Congo, respectively.

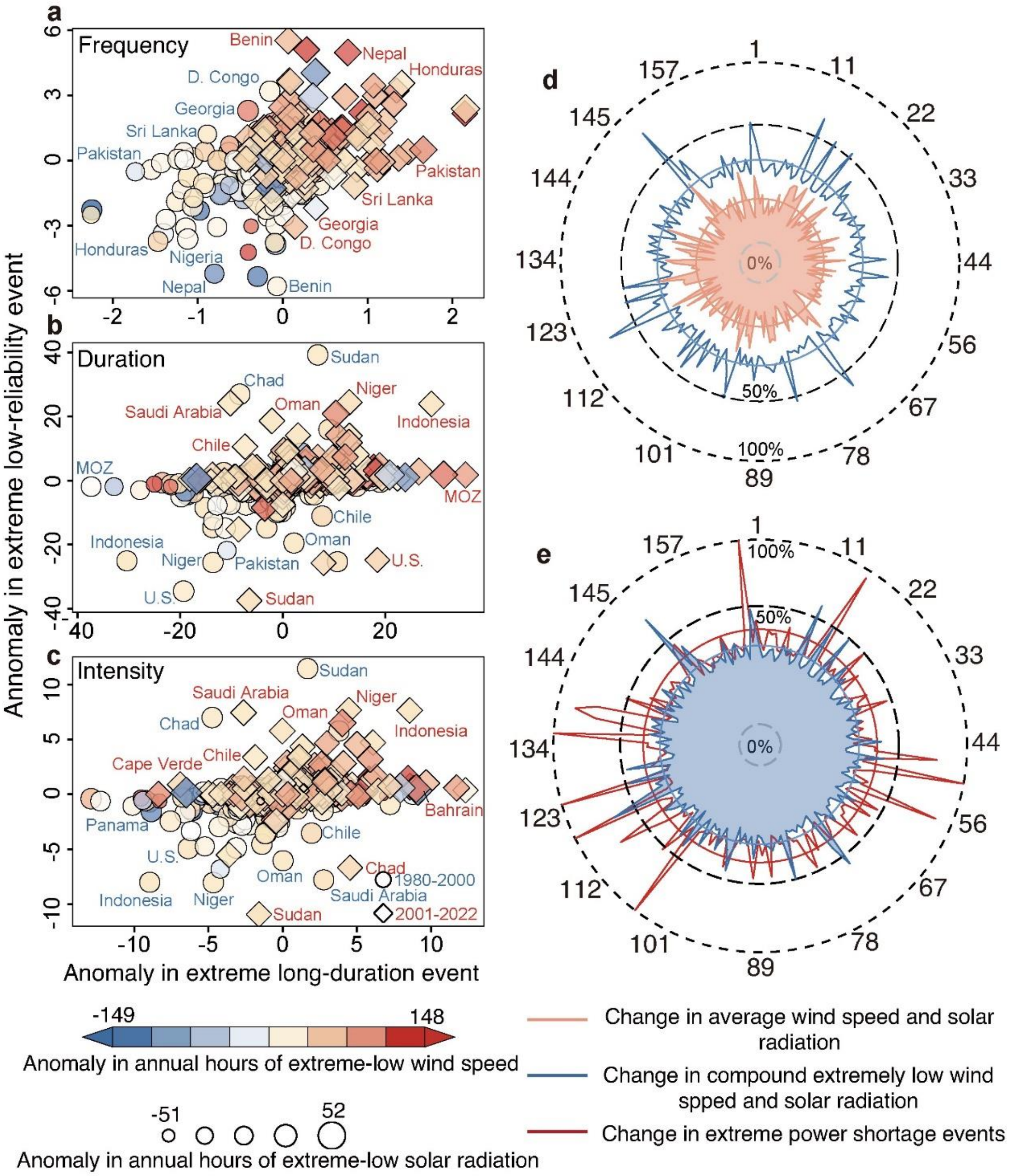

Supplementary Figure 48 | Changes in extreme power shortage events with extremely low wind speed and solar radiation using the wind-solar shares in REMIND mode. a-c, Relationship between anomalies in frequency **(a)**, duration **(b)**, and intensity **(c)** of extreme power shortage events and anomalies in annual hours of extremely low solar radiation and wind speed at hub height for the 178 surveyed countries. **d**, Relationship between change in annual average wind speed and change in annual hours of compound extremely low wind speed & solar radiation between 1980–2000 and 2001–2022. **e**, Relationship between the relative change in annual hours of extremely low wind speed & solar radiation and the relative change in extreme power shortage events between 1980–2000 and 2001–2022. The relative change above 100% is visualized as 100% change. The lines and circles denote individual country's change and the average changes across 178 surveyed countries, respectively. The surrounding digits represent the order number of the 178 countries that are listed in Supplementary Table 7.

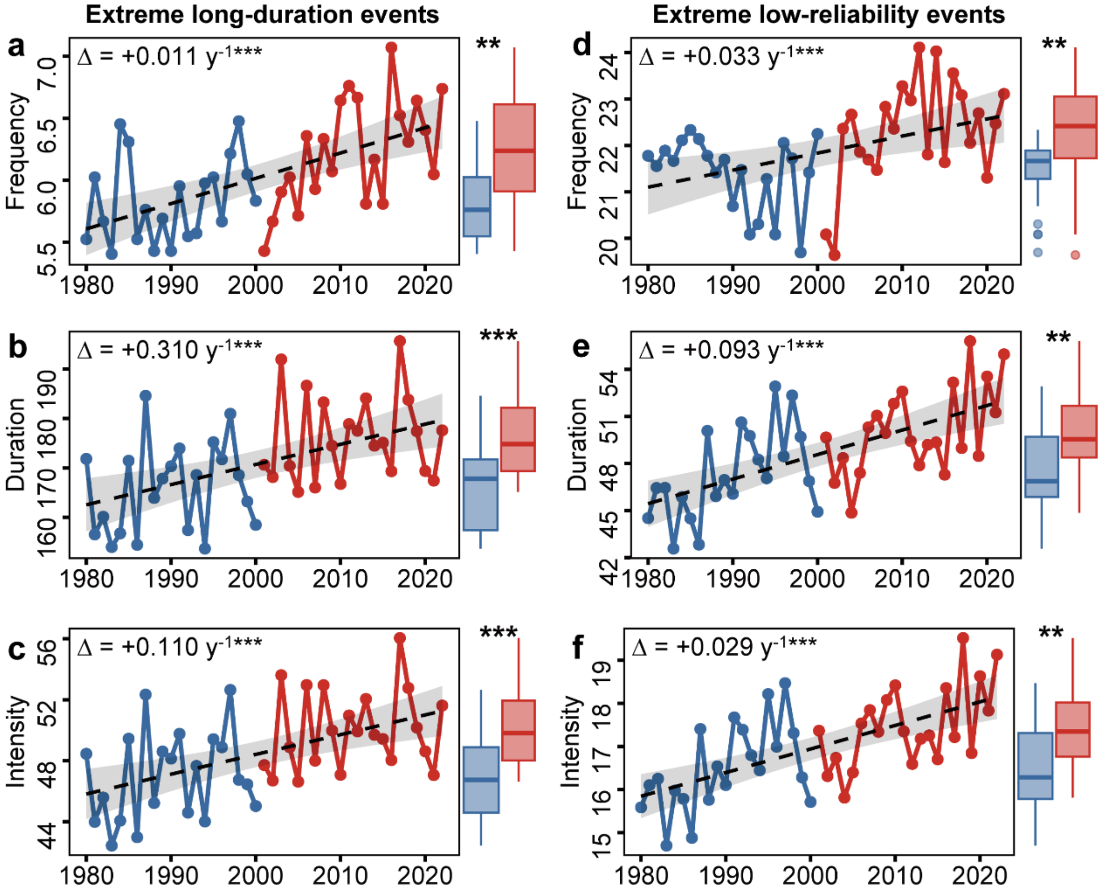

Supplementary Figure 49 | Sensitivity tests for changes in extreme power shortage events based upon projected demand profile by mid-century. a-c, Changes in frequency **(a)**, duration **(b)**, and intensity **(c)** of extreme long-duration events using 43-year hourly climatological variables and projected demand profile by mid-century. **d-f**, Changes in frequency **(d)**, duration **(e)**, and intensity **(f)** of extreme low-reliability events using 43-year hourly climatological variables and projected demand profile by mid-century. The coefficients at the top left of each panels indicate robust Theil–Sen's slopes and their corresponding *P* values, examined by MK test. The right boxplots denote the difference between 1980–2000 (blue) and 2000–2022 (red) examined by ANOVA. *, **, and *** represent the significance under the level of $P<0.05$, $P<0.01$, and $P<0.001$, respectively. Black dashed lines with shading denote linear fitting and the standard deviation of annual average values across 42 countries.

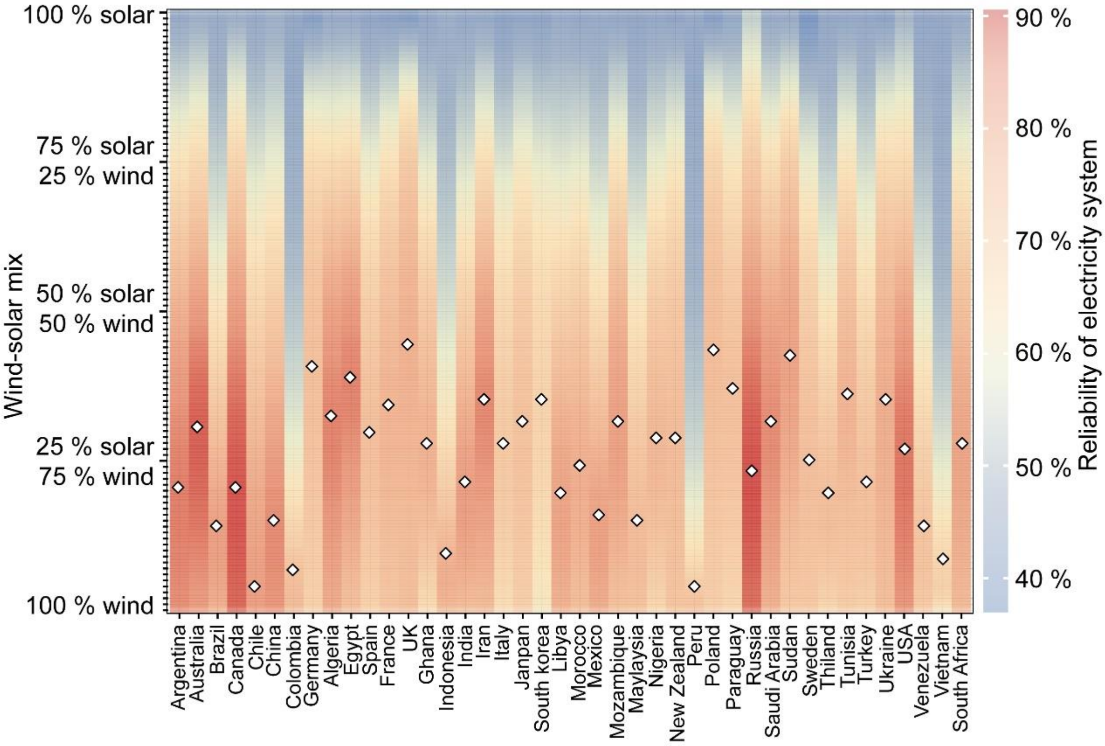

Supplementary Figure 50 | Reliability of wind-solar electricity system by varying wind and solar generation mixes across 42 countries. Shading refers to the 43-year average reliability of electricity system based on varied wind-solar mixes ranging from 0 to 100% (every 1 % change in for wind and solar generation mixes). White points denote the optimal wind-solar mix with the highest reliability for each country.

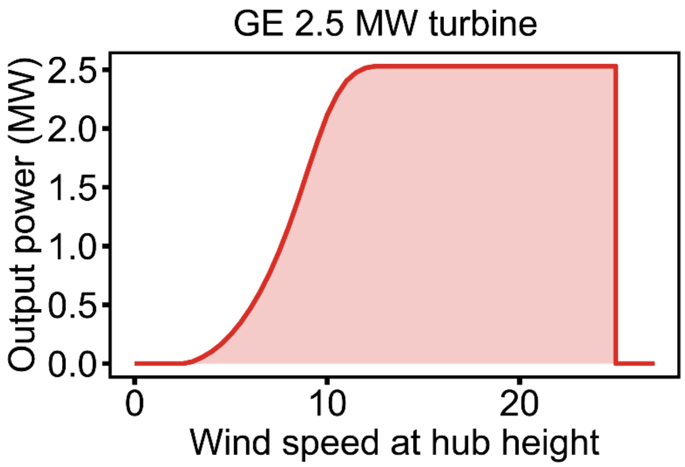

Supplementary Figure 51 | Power curves of onshore GE 2.5 MW wind turbine.

Supplementary Table | 1 The selected 178 major countries/regions

Continents	Involved countries
Oceania	New Zealand, Australia, Kiribati, New Caledonia, Papua New Guinea, Solomon Islands, Vanuatu
Asia	China, India, Japan, South Korea, Indonesia, Saudi Arabia, Iran, Thailand, Vietnam, Pakistan, Malaysia, Philippines, Kazakhstan, United Arab Emirates, Singapore, Bangladesh, Iraq, Uzbekistan, Israel, Kuwait, Hong Kong, Myanmar, Hungary, Qatar, Syria, Sri Lanka, Oman, Bahrain, Jordan, Lebanon, Turkmenistan, Tajikistan, North Korea, Yemen, Cameroon, Kyrgyzstan, Nepal, Afghanistan, Armenia, Laos, Mongolia, Brunei, Bhutan
Europe	France, United Kingdom, Italy, Poland, Turkey, Russia, Spain, Germany, Sweden, Ukraine, Norway, Netherlands, Belgium, Finland, Czech Republic, Austria, Romania, Switzerland, Greece, Portugal, Belarus, Bulgaria, Kosovo, Serbia, Ethiopia, Azerbaijan, Denmark, Ireland, Slovakia, Guatemala, Iceland, Bosnia and Herzegovina, Slovenia, Lithuania, Macedonia, Moldova, Latvia, Luxembourg, Democratic Republic of the Congo, Montenegro, Malta
America	United States, Brazil, Mexico, Argentina, Canada, Venezuela, Colombia, Peru, Chile, Ecuador, Cuba, Puerto Rico, Croatia, Uruguay, Costa Rica, Paraguay, Panama, Bolivia, Trinidad and Tobago, Honduras, Nicaragua, Cyprus, Gabon, Haiti, Jamaica, Guinea, Bahamas, French Guiana, Suriname, Belize, Cape Verde, Antigua and Barbuda
Africa	Egypt, Algeria, Ghana, South Africa, Tunisia, Nigeria, Mozambique, Libya, Sudan, Morocco, Angola, South Sudan, Dominican Republic, Burkina Faso, Kenya, Tanzania, Zambia, Uganda, Zimbabwe, El Salvador, Georgia, Ivory Coast, Albania, Niger, Cambodia, Estonia, Togo, Botswana, Namibia, Chad, Senegal, Rwanda, Mauritius, Mali, Madagascar, Republic of Congo, Malawi, Benin, Somalia, Equatorial Guinea, Mauritania, Western Sahara, Eritrea, Sierra Leone, Swaziland, Guinea-Bissau, Fiji, Guyana, Liberia, Burundi, Lesotho, Djibouti, Gambia, Central African Republic, Seychelles, Sao Tome and Principe

Supplementary Table 2 | Share of wind and solar to the total electricity generation in 2050 based upon IEA

Regions	Share
**World**	59.05%
**North America**	67.45%
United States	72.62%
**Central and South America**	56.36%
Brazil	54.15%
**Europe**	63.13%
European Union	67.47%
**Africa**	56.80%
**Middle East**	53.67%
**Eurasia**	13.40%
Russia	7.48%
**Asia Pacific**	59.41%
China	60.75%
India	71.63%
Japan	38.68%
Southeast Asia	42.49%

Supplementary Table | 3 The selected 42 major countries across the world

Continents	Involved countries
Oceania	New Zealand, Australia
Asia	China, India, Indonesia, Iran, Japan, Malaysia, Saudi Arabia, Thailand, South Korea, Vietnam
Europe	Turkey, Russia, France, Germany, Italy, Sweden, Ukraine, United Kingdom, Spain, Poland
America	United States, Canada, Brazil, Mexico, Venezuela, Argentina, Chile, Colombia, Paraguay, Peru
Africa	Algeria, Egypt, Ghana, Libya, Morocco, Mozambique, Nigeria, South Africa, Sudan, Tunisia

Supplementary Table 4 | Share of wind and solar to the total electricity generation predicted by REMIND model[18,19]

Geographical Zones	Share of wind and solar
Canada, Australia, New Zealand	63.93%
China	67.51%
Europe	87.62%
India	90.67%
Japan	64.70%
Latin America	73.37%
Middle East	91.93%
Non-EU Europe	73.16%
Other Asia	87.77%
Reforming countries	76.48%
Sub-Saharan Africa	84.13%
USA	90.62%
World	82.95%

Supplementary Table 5 | Predicted shares of CSP and PV to the total solar supply by mid-century

Geographical zones	CSP share	PV share
**North America**	8.45%	91.55%
United States	1.94%	98.06%
Mexico	6.64%	93.36%
**Central and South America**	5.73%	94.27%
Brazil	11.08%	88.92%
Argentina	6.70%	93.30%
Chile	0.06%	99.94%
Venezuela	0.30%	99.70%
**Europe**	1.16%	98.84%
Germany	0.79%	99.21%
France	0.69%	99.31%
UK	0.95%	99.05%
Russia	2.46%	97.54%
Austria	0.00%	100.00%
Belgium	0.00%	100.00%
Poland	0.00%	100.00%
Denmark	0.00%	100.00%
Finland	0.00%	100.00%
Greece	1.50%	98.50%
Turkey	2.06%	97.94%
**Africa**	8.45%	91.55%
Morocco	0.05%	99.95%
Angola	0.03%	99.97%
Algeria	0.01%	99.99%
Egypt	0.01%	99.99%
Kenya	0.04%	99.96%
South Africa	19.10%	80.90%
**Middle East**	15.64%	84.36%
Saudi Arabia	22.90%	77.10%
**Asia**	3.22%	96.78%
China	1.21%	98.79%
India	5.63%	94.37%
Japan	0.14%	99.86%
Indonesia	12.13%	87.87%
Pakistan	14.98%	85.02%
**Oceanic**	9.89%	90.11%
Australia	2.87%	97.13%

Supplementary Table 6 | Predicted wind and solar shares to the total wind-solar supply systems by mid-century based on IAMs

Geographical zones		Wind share	Solar share
**World**		48.04%	51.96%
**Africa**		39.78%	60.22%
	South Africa	50.62%	49.38%
**Asia**		45.83%	54.17%
	China	47.37%	52.63%
	India	45.22%	54.78%
	Indonesia	70.64%	29.36%
	Japan	66.48%	33.52%
	Pakistan	45.30%	54.70%
	South Korea	55.59%	44.41%
**Europe**		58.24%	41.76%
	Turkey	56.47%	43.53%
**Middle East**		42.30%	57.70%
**North America**		45.89%	54.11%
	Canada	66.43%	33.57%
	Mexico	55.64%	44.36%
	United States	46.76%	53.24%
	Oceania	64.76%	35.24%
**Eurasia**		45.83%	54.17%
	Russia	59.09%	40.91%
	Azerbaijan	58.24%	41.76%
**Central and South America**		45.89%	54.11%
	Brazil	66.16%	33.84%

Supplementary Table 7 | The order numbers of the selected 178 countries

Order	Country	Order	country	Order	Country	Order	Country
1	Afghanistan	46	Egypt	91	Liberia	136	Saudi Arabia
2	Albania	47	El Salvador	92	Libya	137	Senegal
3	Algeria	48	Equatorial Guinea	93	Lithuania	138	Serbia
4	Angola	49	Eritrea	94	Luxembourg	139	Seychelles
5	Antigua and Barbuda	50	Estonia	95	Macedonia	140	Sierra Leone
6	Argentina	51	Ethiopia	96	Madagascar	141	Singapore
7	Armenia	52	Fiji	97	Malawi	142	Slovakia
8	Australia	53	Finland	98	Malaysia	143	Slovenia
9	Austria	54	France	99	Mali	144	Solomon Islands
10	Azerbaijan	55	French Guiana	100	Malta	145	Somalia
11	Bahamas	56	Gabon	101	Mauritania	146	South Africa
12	Bahrain	57	Gambia	102	Mauritius	147	South Korea
13	Bangladesh	58	Georgia	103	Mexico	148	South Sudan
14	Belarus	59	Germany	104	Moldova	149	Spain
15	Belgium	60	Ghana	105	Mongolia	150	Sri Lanka
16	Belize	61	Greece	106	Montenegro	151	Sudan
17	Benin	62	Guatemala	107	Morocco	152	Suriname
18	Bhutan	63	Guinea	108	Mozambique	153	Swaziland
19	Bolivia	64	Guinea-Bissau	109	Myanmar	154	Sweden
20	Bosnia and Herzegovina	65	Guyana	110	Namibia	155	Switzerland
21	Botswana	66	Haiti	111	Nepal	156	Syria
22	Brazil	67	Honduras	112	Netherlands	157	Tajikistan
23	Brunei	68	Hungary	113	New Caledonia	158	Tanzania
24	Bulgaria	69	Iceland	114	New Zealand	159	Thailand
25	Burkina Faso	70	India	115	Nicaragua	160	Togo
26	Burundi	71	Indonesia	116	Niger	161	Trinidad and Tobago
27	Cambodia	72	Iran	117	Nigeria	162	Tunisia
28	Cameroon	73	Iraq	118	North Korea	163	Turkey

29	Canada	74	Ireland	119	Norway	164	Turkmenistan
30	Cape Verde	75	Israel	120	Oman	165	Uganda
31	Central African Republic	76	Italy	121	Pakistan	166	Ukraine
32	Chad	77	Ivory Coast	122	Panama	167	United Arab Emirates
33	Chile	78	Jamaica	123	Papua New Guinea	168	United Kingdom
34	China	79	Japan	124	Paraguay	169	United States
35	Colombia	80	Jordan	125	Peru	170	Uruguay
36	Costa Rica	81	Kazakhstan	126	Philippines	171	Uzbekistan
37	Croatia	82	Kenya	127	Poland	172	Vanuatu
38	Cuba	83	Kiribati	128	Portugal	173	Venezuela
39	Cyprus	84	Kosovo	129	Puerto Rico	174	Vietnam
40	Czech Republic	85	Kuwait	130	Qatar	175	Western Sahara
41	Democratic Republic of the Congo	86	Kyrgyzstan	131	Republic of Congo	176	Yemen
42	Denmark	87	Laos	132	Romania	177	Zambia
43	Djibouti	88	Latvia	133	Russia	178	Zimbabwe
44	Dominican Republic	89	Lebanon	134	Rwanda		
45	Ecuador	90	Lesotho	135	Sao Tome and Principe		

References:

1. Tong, D. *et al.* Geophysical constraints on the reliability of solar and wind power worldwide. *Nat. Commun.* **12**, 6146 (2021).
2. Feron, S., Cordero, R. R., Damiani, A. & Jackson, R. B. Climate change extremes and photovoltaic power output. *Nat. Sustain.* **4**, 270–276 (2021).
3. Gao, M. *et al.* Secular decrease of wind power potential in India associated with warming in the Indian Ocean. *Sci. Adv.* **4**, eaat5256 (2018).
4. Vautard, R., Cattiaux, J., Yiou, P., Thépaut, J. N. & Ciais, P. Northern Hemisphere atmospheric stilling partly attributed to an increase in surface roughness. *Nat. Geosci.* **3**, 756–761 (2010).
5. Pryor, S. C. *et al.* Wind speed trends over the contiguous United States. *J. Geophys. Res. Atmos.* **114**, 1–18 (2009).
6. Zeng, Z. *et al.* A reversal in global terrestrial stilling and its implications for wind energy production. *Nat. Clim. Chang.* **9**, 979–985 (2019).
7. Karnauskas, K. B., Lundquist, J. K. & Zhang, L. Southward shift of the global wind energy resource under high carbon dioxide emissions. *Nat. Geosci.* **11**, 38–43 (2018).
8. Rei, C. & Janni, Y. Human-induced weakening of the Northern Hemisphere tropical circulation. *Nature* (2023) doi:10.1038/s41586-023-05903-1.
9. Gernaat, D. E. H. J. *et al.* Climate change impacts on renewable energy supply. *Nat. Clim. Chang.* **11**, 119–125 (2021).
10. Jerez, S. *et al.* The impact of climate change on photovoltaic power generation in Europe. *Nat. Commun.* **6**, 10014 (2015).
11. Li, X., Mauzerall, D. L. & Bergin, M. H. SI Global reduction of solar power generation efficiency due to aerosols and panel soiling. *Nat. Sustain.* **3**, 720–727 (2020).
12. Li, X., Wagner, F., Peng, W., Yang, J. & Mauzerall, D. L. Reduction of solar photovoltaic resources due to air pollution in China. *Proc. Natl. Acad. Sci. U. S. A.* **114**, 11867–11872 (2017).
13. Sweerts, B. *et al.* Estimation of losses in solar energy production from air pollution in China since 1960 using surface radiation data. *Nat. Energy* **4**, 657–663 (2019).
14. Yao, F. & Palmer, P. I. Source Sector Mitigation of Solar Energy Generation Losses Attributable to Particulate Matter Pollution. *Environ. Sci. Technol.* **56**, 8619–8628 (2022).
15. EIA. International electricity demand data. *U.S. Energy Inf. Adm.* (2023).
16. Byers, E., Krey, V., Kriegler, E. & Riahi, K. AR6 Scenarios Database hosted by IIASA. *Int. Inst. Appl. Syst. Anal.* (2022) doi:10.5281/zenodo.5886911.
17. Crook, J. A., Jones, L. A., Forster, P. M. & Crook, R. Climate change impacts on future photovoltaic and concentrated solar power energy output. *Energy Environ. Sci.* **4**, 3101–3109 (2011).
18. Luderer, G. *et al.* Impact of declining renewable energy costs on electrification in low-emission scenarios. *Nat. Energy* **7**, 32–42 (2022).
19. Dietrich, J. P. *et al.* MAgPIE 4-a modular open-source framework for modeling global land systems. *Geosci. Model Dev.* **12**, 1299–1317 (2019).
