# Supplementary material for: Climate change impacts on the extreme power shortage events of wind-solar supply systems worldwide during 1980–2022
Source: Nat Commun. 2024 Jun 18;15:5225. doi: 10.1038/s41467-024-48966-y (PMC11189577; doi:10.1038/s41467-024-48966-y)
Supplement: Supplementary file 3 — Reporting Summary [file 41467_2024_48966_MOESM3_ESM.pdf]

nature portfolio

Corresponding author(s): Dan Tong

Last updated by author(s): 27/04/24

Reporting Summary

Nature Portfolio wishes to improve the reproducibility of the work that we publish. This form provides structure for consistency and transparency in reporting. For further information on Nature Portfolio policies, see our Editorial Policies and the Editorial Policy Checklist.

Statistics

For all statistical analyses, confirm that the following items are present in the figure legend, table legend, main text, or Methods section.

n/a	Confirmed	
☐	☒	The exact sample size (*n*) for each experimental group/condition, given as a discrete number and unit of measurement
☐	☒	A statement on whether measurements were taken from distinct samples or whether the same sample was measured repeatedly
☐	☒	The statistical test(s) used AND whether they are one- or two-sided<br>*Only common tests should be described solely by name; describe more complex techniques in the Methods section.*
☐	☒	A description of all covariates tested
☐	☒	A description of any assumptions or corrections, such as tests of normality and adjustment for multiple comparisons
☐	☒	A full description of the statistical parameters including central tendency (e.g. means) or other basic estimates (e.g. regression coefficient) AND variation (e.g. standard deviation) or associated estimates of uncertainty (e.g. confidence intervals)
☐	☒	For null hypothesis testing, the test statistic (e.g. *F*, *t*, *r*) with confidence intervals, effect sizes, degrees of freedom and *P* value noted<br>*Give P values as exact values whenever suitable.*
☒	☐	For Bayesian analysis, information on the choice of priors and Markov chain Monte Carlo settings
☐	☒	For hierarchical and complex designs, identification of the appropriate level for tests and full reporting of outcomes
☐	☒	Estimates of effect sizes (e.g. Cohen's *d*, Pearson's *r*), indicating how they were calculated

Our web collection on statistics for biologists contains articles on many of the points above.

Software and code

Policy information about availability of computer code

Data collection	The code for collecting ERA5 climatic reanalysis data can be acquired from CDS Toolbox (https://cds.climate.copernicus.eu/toolbox/doc/index.html).
Data analysis	The code for estimating hourly wind and solar power capacity factors used on reanalysis data is available on https://github.com/carnegie/Create_Wind_and_Solar_Resource_Files.

For manuscripts utilizing custom algorithms or software that are central to the research but not yet described in published literature, software must be made available to editors and reviewers. We strongly encourage code deposition in a community repository (e.g. GitHub). See the Nature Portfolio guidelines for submitting code & software for further information.

Data

Policy information about availability of data

All manuscripts must include a data availability statement. This statement should provide the following information, where applicable:

- Accession codes, unique identifiers, or web links for publicly available datasets
- A description of any restrictions on data availability
- For clinical datasets or third party data, please ensure that the statement adheres to our policy

Hourly reanalysis climatological fata for the calculation of wind and solar capacity factors can be accessed from MERRA-2 (https://gmao.gsfc.nasa.gov/reanalysis/MERRA-2/) and ERA5 (https://cds.climate.copernicus.eu/cdsapp#!/home). Future supply share of CSP and photovoltaic solar are available from International

Institute for Applied Systems Analysis (IIASA; https://data.ece.iiasa.ac.at/ar6/). Raw data for extreme power shortage events for individual countries is provided on Zenodo (https://zenodo.org/records/11066289). Source data are provided with this paper.

Research involving human participants, their data, or biological material

Policy information about studies with human participants or human data. See also policy information about sex, gender (identity/presentation), and sexual orientation and race, ethnicity and racism.

Reporting on sex and gender	This information has not been collected.
Reporting on race, ethnicity, or other socially relevant groupings	This manuscript dose not involve race, ethnicity, or other socially relevant groupings.
Population characteristics	This manuscript dose not involve population characteristics.
Recruitment	This manuscript dose not involve recruitment
Ethics oversight	This manuscript dose not involve ethics oversight

Note that full information on the approval of the study protocol must also be provided in the manuscript.

Field-specific reporting

Please select the one below that is the best fit for your research. If you are not sure, read the appropriate sections before making your selection.

☐ Life sciences ☐ Behavioural & social sciences ☒ Ecological, evolutionary & environmental sciences

For a reference copy of the document with all sections, see nature.com/documents/nr-reporting-summary-flat.pdf

Ecological, evolutionary & environmental sciences study design

All studies must disclose on these points even when the disclosure is negative.

Study description	We leverage 43-year (i.e., 1980–2022) hourly Modern-Era Retrospective analysis for Research and Application Version-2 (MERRA-2) reanalysis climatological data to derive area-weighted wind and solar capacity factors (assuming dual-axis solar tracking system) for individual countries. Then, we estimate the hourly electricity supply in wind-solar systems, assuming the reliability-optimized wind/solar generation ratio and the projected install capacity by mid-century. The resulting hourly electricity supply and actual/predicted hourly demand from a single recent year are applied to identify two types of defined extreme power shortage events over the past 43 years.
Research sample	We present some of our results for 42 major countries across the world.
Sampling strategy	We present some results for 42 major countries (Figs. 4–5). These countries were chosen based on power demand and regional representation. To be specific, we selected the top 10 countries with the highest electricity demand for individual continents (i.e., Asia, Europe, Africa, and America), apart from Oceania in which only two countries (i.e., Australia and New Zealand) were chosen (Supplementary Table 2). The resulting 42 major countries represented ~87% of the total power demand around the world15. Our study also shows the corresponding results (Supplementary Figures 16–17) across the global 178 counties (Supplementary Table 1) that covers ~99% of power demand across the globe15, and the expanded results do not alter our main conclusions.
Data collection	We obtained hourly climatological variables from MERRA-2 (https://gmao.gsfc.nasa.gov/reanalysis/MERRA-2/) and ERA5 (https://cds.climate.copernicus.eu/cdsapp#!/home) reanalysis product. We obtained hourly electricity demand data at country level using Tong et al., method that integrates datasets derived from public power system datasets, previous studies, as well as government and electricity market websites. With regard to countries unavailable on the real-world demand data, we introduced Toktarova et al. hourly electricity demand44 dataset that was projected using actual demand load profiles, socio-economic variables, as well as climatological factors. uture supply share of CSP and photovoltaic solar are available from International Institute for Applied Systems Analysis (IIASA; https://data.ece.iiasa.ac.at/ar6/).
Timing and spatial scale	The hourly reanalysis data across the world had been collected from 01.09.2022 to 24.02.2023. The hourly electricity demand data across the world had been collected from 01.09.2022 to 18.11.2022. Future supply share of CSP and photovoltaic solar had been collected from 01.12.2023 to 29.12.2023.
Data exclusions	We excluded data future supply share of CSP in regional scale when more detailed national data is available.
Reproducibility	All the results can be reproduced using the same data and method in the manuscript.
Randomization	It is not relevant to this study. We estimate the extreme power shortage events of wind-solar system across the world since 1980s, and it does involve randomization.

Blinding	It is not relevant to this study. Our study dose not involve ecological, evolutionary, social, and life sciences.

Did the study involve field work? ☐ Yes ☒ No

Reporting for specific materials, systems and methods

We require information from authors about some types of materials, experimental systems and methods used in many studies. Here, indicate whether each material, system or method listed is relevant to your study. If you are not sure if a list item applies to your research, read the appropriate section before selecting a response.

Materials & experimental systems

n/a	Involved in the study
☒	☐ Antibodies
☒	☐ Eukaryotic cell lines
☒	☐ Palaeontology and archaeology
☒	☐ Animals and other organisms
☒	☐ Clinical data
☒	☐ Dual use research of concern
☒	☐ Plants

Methods

n/a	Involved in the study
☒	☐ ChIP-seq
☒	☐ Flow cytometry
☒	☐ MRI-based neuroimaging

Plants

Seed stocks	It is not relevant to this study.
Novel plant genotypes	It is not relevant to this study.
Authentication	It is not relevant to this study.
